# A cross-cultural examination of bi-directional mentalising in autistic and non-autistic adults

**DOI:** 10.1186/s13229-025-00659-z

**Published:** 2025-05-14

**Authors:** Bianca A. Schuster, Y. Okamoto, T. Takahashi, Y. Kurihara, C. T. Keating, J. L. Cook, H. Kosaka, M. Ide, H. Naruse, C. Kraaijkamp, R. Osu

**Affiliations:** 1 School of Human Sciences, Waseda University, Tokorozawa, Japan; 2 Waseda Institute for Advanced Study, Waseda University, Tokyo, Japan; 3 Department of Cognition, Emotion, and Methods in Psychology, University of Vienna, Vienna, Austria; 4 Department of Sports Science, Faculty of Health and Sports Science, Juntendo University, Tokyo, Japan; 5 Centre for Human Brain Health and School of Psychology, University of Birmingham, Birmingham, UK; 6 Department of Experimental Psychology, University of Oxford, Oxford, UK; 7 University of Fukui, Fukui, Japan; 8 National Rehabilitation Center for Persons With Disabilities, Tokorozawa, Japan

**Keywords:** Autism, Mentalising, Theory of mind, Cross-cultural, Cross-neurotype, Collectivist, Individualist, Japan, UK, Double empathy, Movement differences

## Abstract

**Background:**

So-called ‘mismatch accounts’ propose that, rather than arising from a socio-cognitive deficit present in autistic people, mentalising difficulties are the product of a mismatch in neurotype between interaction partners. Although this idea has grown in popularity over recent years, there is currently only limited empirical evidence to support mismatch theories. Moreover, the social model of disability such theories are grounded in demands a culturally situated view of social interaction, yet research on mentalising and/or autism is largely biased towards Western countries, with little knowledge on how successful mentalising is defined differently, and how tools to assess socio-cognitive ability compare, across cultures.

**Methods:**

Using a widely employed mentalising task—the animations task—, the current study investigated and compared the bi-directional mentalising performance of British and Japanese autistic and non-autistic adults and assessed observer-agent kinematic similarity as a potential dimension along which mismatches may occur between neurotypes. Participants were asked to depict various mental state- and action-based interactions by moving two triangles across a touch-screen device before viewing and interpreting stimuli generated by other participants.

**Results:**

In the UK sample, our results replicate a seminal prior study in showing poorer mentalising abilities in non-autistic adults for animations generated by the autistic group. Crucially, the same pattern did not emerge in the Japanese sample, where there were no mentalising differences between the two groups.

**Limitations:**

Limitations of the current study include that efforts to match all samples within and across cultures in terms of IQ, gender, and age were not successful in all comparisons, but control analyses suggest this did not affect our results. Furthermore, any performance differences were found for both the mental state- and action-based conditions, mirroring prior work and raising questions about the domain-specificity of the employed task.

**Conclusions:**

Our results add support for a paradigm shift in the autism literature, moving beyond deficit-based models and towards acknowledging the inherently relational nature of social interaction. We further discuss how our findings suggest limited cultural transferability of common socio-cognitive measures rather than superior mentalising abilities in Japanese autistic adults, underscoring the need for more cross-cultural research and the development of culturally sensitive scientific and diagnostic tools.

**Supplementary Information:**

The online version contains supplementary material available at 10.1186/s13229-025-00659-z.

## Introduction

Social communication difficulties form one of the core characteristics of autism spectrum conditions. Within the socio-cognitive literature, theories that postulate a ‘deficit’ in Theory of Mind (ToM, the capacity to understand other people by ascribing mental states, such as beliefs, desires, or intentions, to them [1]) within autistic people have long represented a popular explanation for such communication challenges (e.g., see references [2–4] for a critical review of the pervasiveness of this claim). Crucially, this social deficit perspective places the burden to conform to the ‘social norm’ on autistic people, leading to stigma and consequently to camouflaging behaviour [4–6], both of which have been associated with poor mental health outcomes, including increased anxiety, depression, and suicidality [7–11]. Besides being potentially harmful to individuals, a growing body of work has recently theoretically and empirically challenged ToM deficit models of autism, arguing that the theory lacks agreement on some key concepts (such as the definition of a ‘mental state’ [12]), and that existing evidence lacks specificity and replication [4]. Contrasting with social deficit views, the idea that social communication difficulties between neurotypical and autistic partners may be bi-directional has gained increasing attention in recent years, gradually shifting how we conceptualise autism and atypical social cognition away from a social deficit (or ‘medical’) model to a social model of disability. The social model views autism as a socially constructed disability, and thus, the challenges autistic people face arise from conditions given by the specific social context they live in [13]. Among prominent attempts to re-conceptualise social interaction difficulties in autism are the ‘double empathy problem’ [14, 15] and the ‘dialectical misattunement hypothesis’ [16] (reviewed in detail in Davis & Crompton [17]). Both concepts challenge the long-held idea that social interaction difficulties stem from a socio-cognitive deficit solely present in the autistic population and suggest they may rather arise from a mismatch in perspective (where perspective is broadly defined and can range from cognitive processing styles to values) between neurotypes. By relocating the pressure to ensure the success of social interactions from just one group of people to all agents involved, this new perspective has the potential to fundamentally change how we view autism and support autistic individuals. With such potential for improving autistic individuals’ lives, mismatch accounts have increasingly been acknowledged by both the scientific field and society in general. Yet, theoretical and empirical issues with mismatch accounts have been raised [12, 18]. The double empathy problem, in particular, has been argued to be underspecified, generating vague predictions which are difficult to falsify. As a consequence, systematic empirical investigations testing the theory are scarce, and the extant evidence is confounded by methodological limitations, making it difficult to link results to clearly defined underlying socio-cognitive processes.

So-called animations tasks, requiring participants to interpret short videos of interacting triangles by ascribing mental states to them, have extensively been used to measure differences in socio-cognitive ability between autistic and non-autistic people [19–21]. Traditionally, autistic individuals have been found to provide fewer and/or less appropriate mental state descriptions (where ‘appropriateness’ was defined as high agreement with the word that an animation was originally intended to depict) than controls in these tasks [20–25], a finding which was thus far interpreted as evidence for a deficit in spontaneous mentalising within the autistic group. One recent study [26], however, explored the possibility that performance differences between autistic and non-autistic participants observed in previous studies may have emerged because the stimuli in these studies had been created by *non-autistic* experimenters. Edey and colleagues asked autistic and non-autistic adults to first generate their own animations of mental state interactions (such as one triangle ‘surprising’ the other triangle) and to subsequently interpret animations created by both their own- and the other group. In addition to replicating previous studies by showing that autistic participants struggled to interpret those animations made by non-autistic participants, their findings suggested that non-autistic individuals exhibit similar difficulties in interpreting depictions of social interactions when these were created by autistic people, thus providing partial support for the double-empathy theory. Since mental states in animations tasks are almost exclusively conveyed via movement cues, the authors attributed the observed bi-directional mentalising difficulties to differences in the way the two groups used their own movement kinematics to depict and understand the displayed mental states. A growing body of empirical work [27–31] supports the idea that human observers use learned associations between sensory-motor representations of their own movements and internal states to infer the mental states underlying others’ movements. This line of evidence is corroborated further by studies which show that higher overlap in the lower-level features of one’s own and observed movements facilitates this inference [32–34]. Importantly, Edey et al. and a large number of other studies have shown that autistic people move differently to their non-autistic peers, for instance, exhibiting higher jerk (i.e., higher rates of acceleration and deceleration) in upper limb [26, 35–37] and walking movements [38–40], with differences present early in development. In Edey et al.’s study, relative to non-autistic participants, the autistic group used jerkier movements to depict all four mental state interactions. Thus, in the context of traditional animations tasks, mentalising difficulties may have arisen from the fact that autistic individuals have developed different internal representations of the kinds of movements they associate with particular mental states. In other words, autistic people may have struggled to interpret animations created by non-autistic participants because they themselves would have used different movements to represent the same mental state. However, no study to date has directly tested this hypothesis.

Notably, similarly to the perspective mismatch among different neurotypes, different cultures have divergent conceptualisations of what constitutes ‘successful’ social interaction. What is deemed as atypical social behaviour is largely culturally dependent [41], with culturally specific social norms, values, and expectations for social roles dictating what type of behaviour is socially appropriate in a given context. For example, while omitting eye contact during conversation is considered a hallmark of social dysfunction in most Western cultures, the same behaviour may be deemed entirely appropriate in other cultures. In Japan, for instance, eye contact is deemed less important for social interaction, and direct gaze is perceived as unpleasant or unapproachable [42]. Yet, our current understanding of socio-cognitive ability and what constitutes aberrations from the ‘social norm’ is based on research that is highly biased towards Western cultures [43]. Crucially, although evidence is limited and inconclusive, the extant literature examining socio-cognitive differences between cultures suggests that findings from Western studies may not be transferrable across cultures. For example, relative to their peers from individualist cultures, multiple cross-cultural studies indicate delayed onset of ‘false belief’ understanding (i.e., the understanding that other people’s beliefs about reality may contrast with one’s own beliefs and reality itself) and divergent trajectories in the development of ToM in children from collectivist cultures (e.g., [44–47], for a review see [48]). Koelkebeck et al., who employed a version of the animations task to compare mentalising between Japanese and Caucasian adults, found no cross-cultural differences in behaviour but did identify differences in the brain regions recruited during mentalising between the two groups [49]: Compared to Caucasian individuals, Japanese participants showed less task-related activation in the medial prefrontal cortex (mPFC), a core structure of the so-called mentalising network [50]. It is worth noting that the Caucasian participants in the latter study were living in Japan, which may have contributed to the lack of behavioural differences. Observed cultural differences in behavioural and neural correlates of socio-cognitive development have been attributed in part to methodology. That is, they emerge predominantly in studies employing explicit, as opposed to implicit measures of ToM (although there is some debate around the reliability and validity of implicit ToM tasks altogether, e.g., see references [12, 51]). Cultural differences may also arise from differences in cultural attribution styles, linguistic variables and parenting characteristics [48]. For instance, it has been argued that cultural beliefs may drive the extent to which individuals use mental states, rather than social norms or roles, to explain observed behaviour, and consequently that cultures which prioritise the group over the individual focus more on the latter [52, 53]. Additionally, there may be other culturally relevant components driving cross-cultural differences in mentalising, such as the extent to which one’s body movements are used to communicate emotions and mental states. Cultural norms and conventions around bodily expressions, such as attitudes towards the open expression of emotions [54], may influence the extent to which individuals modulate their own movement to communicate internal states and, ultimately, the extent to which they use others’ movements to mentalise [55]. Indeed, it has been suggested that culture shapes the associations formed between different concepts and sensorimotor experiences [56]. For instance, while it is well-established that Western participants use temporal and spatial movement patterns as cues when inferring others’ intentions and mental states [32–34, 57, 58], a lower tendency among Japanese individuals to express their internal states in body movements [59] may indicate that they rely on cues unrelated to body movement when decoding others’ internal states.

In summary, research findings based on Western samples dominate the global understanding of what constitutes ‘typical' and atypical social communication, while we currently do not have a good understanding of the extent to which these results apply to other, non-Western cultures [60]. This is reflected in the fact that one of the gold-standard diagnostic tools for autism, the Autism Diagnostic Observation Scale (ADOS-2 [61])—a measure which was initially developed based on Western samples—is widely used alongside the DSM-V to diagnose autism in non-Western cultures such as Japan (e.g., see Saito et al. [62]). This is problematic because at least some of the items are not directly applicable to other cultures. For instance, items assessing eye contact, as well as the frequency and timing of gestures, show high cross-cultural variation [63]. Other Western-based diagnostic tools, such as the Autism Spectrum Quotient (AQ [64]), have similarly shown lower applicability to Japanese and other non-Western samples [65]. The potential lack of cultural transferability of research findings, as well as questionable cultural sensitivity of commonly used diagnostic tools, may at least partially be responsible for existing discrepancies in autism prevalence and differences in age at diagnosis between the different cultures [66]. Yet, although calls for situating the diagnosis of autism within the cultural context of the individual were first raised over a decade ago [67], so far, little effort has been made to address issues of cultural transferability.

By comparing mentalising in British and Japanese autistic and non-autistic adults, the current pre-registered (https://osf.io/caqfk) study thus addresses two primary questions: Is the finding of bi-directional mentalising difficulties between autistic and non-autistic adults (I) stable (i.e., will it replicate in the British sample) and (II) transferable to non-Western cultures (i.e., will the same pattern emerge in the Japanese sample)? Our secondary objective related to investigating the degree to which observer-agent kinematic differences represent one dimension along which mentalising mismatches may occur between neurotypes. We had several specific hypotheses relating to these questions, which are specified in Table 1. To test these hypotheses, British and Japanese autistic and non-autistic participants were asked to complete a version [33, 34] of the animations task wherein they first created their own mental- and non-mental state animations before viewing and interpreting animations generated by all other participants. Our results replicate the previously found bi-directional mentalising difficulties between autistic and non-autistic people in the UK- but not the Japanese sample, thus raising important questions about the cultural transferability of our current knowledge of typical and atypical social cognition.

**Table 1:** Summary of all pre-registered (https://osf.io/caqfk) hypotheses

Hypothesis	Description	Relevant prior literature
1	Japanese non-autistic individuals will show difficulties in interpreting animations created by Japanese autistic participants to an extent comparable to the performance of British non-autistic participants when interpreting animations made by British autistic participants	Edey et al. [26]
2	Replicating a previous study [26], British non-autistic participants will show poorer performance when interpreting animations created by British autistic participants	Edey et al. [26]
3	There will be no differences in performance between the two autistic groups when rating animations created by autistic participants of their own, relative to the respective other culture	Chevallier et al. [68], Izuma et al. [69], Large et al. [70]
4	UK autistic participants will show no 'own group bias' when rating animations of other UK autistic people, as was found in Edey et al. [26], and will thus show comparable performance when rating animations created by UK autistic and non-autistic individuals	Edey et al. [26]
5	The more similar a British observer's own movement kinematics are to the movement kinematics of a given animation, the better they will be at accurately labelling it, regardless of whether they have an ASD diagnosis or not	De Marco et al. [32], Schuster et al. [33], Schuster et al. [34]
6	Animations created by autistic participants of both cultures will exhibit higher mean jerk than animations created by Japanese or British non-autistic participants	Edey et al. [26], Cook et al. [36], Yang et al. [35]
7	Movement kinematics will play a lesser role in the communication of mental states among Japanese people, and therefore the effect of movement differences on mental state attribution accuracy will be less strong in this culture compared to British participants	Lillard [52], Heyes and Frith [53], Matsumoto et al. [54]

## Methods

### Participants

Forty-eight Japanese (25 autistic, 23 non-autistic) and 49 UK-based adults (25 autistic, 24 non-autistic; for full sample details see Table 2) completed both parts of the study, resulting in 97 full datasets forming the basis for all following analyses. Sample size was determined based on a power analysis for hypotheses one and two (using an application for random effects designs, see [71] and https://jakewestfall.shinyapps.io/crossedpower/), which indicated that a total of 30 participants per culture (15 autistic, 15 non-autistic) are sufficient to detect a medium-sized effect of d = 0.5 with 80% power. In Japan, autistic participants were recruited from clinical services and non-autistic participants were recruited from an established university participant database. In the UK, both autistic and non-autistic participants were recruited via university mailing lists as well as community advertising in different social media channels. To be classified as members of a given culture group, participants were required to have lived in the respective country for a minimum of 10 years (given a lack of evidence-based guidelines, this number was chosen as an arbitrary cut-off value). All autistic participants had a clinical diagnosis of autism or autism spectrum disorder. Both (Japanese and British) autistic groups had higher autism quotient (AQ) scores [64] than the non-autistic participants (see Table 2), and the mean AQ scores in both autistic and non-autistic groups were highly comparable to that found in large population samples (e.g., Ruzich et al. [72]; Wakabayashi et al. [73]). Out of the 25 autistic UK participants, three individuals had a co-occurring learning disability. None of the Japanese autistic participants were diagnosed with a learning disability. All participants gave written informed consent and received either money (£10 per hour/¥ 4000 per session) or course credit for their participation. Experimental procedures were approved by the University of Birmingham Research Ethics Committee (ERN_16-0281AP5C) and the Ethics Committee on Human Research of Waseda University (2021-021 (2)) and performed in accordance with the WMA Declaration of Helsinki [74].

**Table 2:** Demographic data for Japanese and UK, autistic and non-autistic samples. JP = Japanese culture, UK = British culture. FSIQ = Full-scale IQ; FSIQ was assessed with the Wechsler Adult Intelligence Scale (WAIS)-IV [75] in Japanese participants, approximations of FSIQ were derived from Wechsler Abbreviated Scale of Intelligence (WASI)-II [76] scores in UK participants. Average time in culture is displayed as average proportion of lifetime (years lived in culture divided by age)

Test	UK			JP		
	Autistic (n = 25)	Non-autistic (n = 24)	Group comparison	Autistic (n = 25)	Non-autistic (n = 23)	Group comparison
Gender F/M/O	13/11/1	15/9/0	–	9/16/0	7/16/0	–
Age M (SD)	31.64 (9.68)	24.33 (7.02)	*p* = .004	29.12 (6.00)	27.39 (8.16)	*p* = .405
Average time in culture	93.36%	87.99%	–	100%	99.55%	–
FSIQ M (SD)	108.29 (18.74, [n = 24])	114.13 (9.94, [n = 23])	*p* = .198	105.12 (13.12, [n = 25])	113.44 (8.38, [n = 18])	*p* = .023
AQ M (SD)	34.48 (8.12, [n = 23])	13.25 (4.87, [n = 23])	*p* < .001	34.64 (6.1)	16.73 (5.63, [n = 22])	*p* < .001

Cross-cultural comparisons (UK vs JP)		
	Autistic	Non-autistic
Age	*p* = .248	*p* = .175
FSIQ	*p* = .488	*p* = .816
AQ	*p* = .775	*p* = .021

### Tasks and procedure

#### Animations task

We used an adaptation of a classical mentalising task—the animations task—previously introduced in Schuster et al. [33], where participants were first asked to create their own short videos of interacting triangles to depict so-called mental state and non-mental state words before interpreting videos that had been generated by their fellow participants. Within each mental state category, three words describing an interaction were chosen in equivalence to the words used in two seminal studies by Abell et al. [77] and Edey et al. [26] and multiple following studies [21–23, 78], and adjusted for semantic equivalence across cultures: Mental state: arguing, surprising, teasing (口論する, 驚かす, からかう); non-mental state: following, searching, dancing (ついていく, 探す, 踊る). The key distinction between the two conditions is that the mental-state (ToM) animations entail propositional attitudes wherein one agent intends to cause or act upon a particular mental state in the other (in prior research referred to as ‘ToM’ interactions), while non-mental state (also called ‘goal-directed’) animations involve reciprocal interaction without the intention to cause/act upon the other triangle’s mental state and thus do not require such causal inference [79]. This distinction is corroborated by prior research where the latter have been shown to consistently elicit lower levels of spontaneous attributions of intentionality than the mental state animations [21–23].

On two separate days, participants first generated one animation for each of the six words by moving two triangles around the touchscreen of a Galaxy A7 tablet. To evaluate possible motor contributions to (mental-) state inference, positional data of individuals’ bi-manual finger movements was recorded at 60 Hz. On average 3.4 (max. 8) months later, participants viewed and rated a total of 144 animations which were pseudo-randomly selected from each of the four generator pools (6 animations per animated word, generator group and generator culture [UK, Japan, autistic, non-autistic]). After viewing each animation, participants indicated on visual-analogue scales ranging from 0 to 100 how likely they thought the animation last viewed depicted each of the six possible words. As in our two previous studies [33, 34], accuracy for each trial was calculated by subtracting the mean rating for all non-target words from the rating for the target word. Thus, a positive score indicates that the target word (e.g., surprising) was rated higher than the average of all non-target words (e.g., arguing, teasing, following, searching, dancing) with higher positive accuracy scores reflecting better discrimination between target and non-target words and lower or negative accuracy scores representing high confusion between scales.

### Statistical analyses

All data were processed in MATLAB R2023b [80] and analysed with Bayesian mixed-effects models using the brms [81] package in R [82]. Prior to model building, any continuous predictors were normalised (z-standardised) to allow comparisons between individual estimates. We used a standard analysis package (brms [81]) to assess the evidence for alternative models using a leave-one-out-cross-validation scheme (using the LOO [83] function). Crucially, using plausible (mildly informative) priors over random effects, this kind of analysis eludes a point null hypothesis—and allows us to specify, with a certain confidence, whether an effect was present or absent. This confidence is reflected by the 95% credible intervals (CrIs, the Bayesian analogue of the classical confidence interval, with the exception that probability statements can be made based on CrIs [84]), as well as the posterior probability that a certain effect ($E\mu$) is different from zero ($P(E\mu <0)$ or $P(E\mu >0)$). Consequently, for all relevant model parameters, we report expected values under the posterior distribution and their CrIs, as well as their posterior probabilities. In line with Franke & Roettger [85], we conclude there is compelling evidence for an effect if its posterior probability $P\left(E\mu \ne 0\right)$ is close to 1. Post-hoc contrasts were tested using the emmeans package [86]. Evidence for null effects was additionally evaluated using the Savage-Dickey density ratio as an approximation to the Bayes Factor and thus quantifies the evidence for a model with- over a model without a given effect. We used generic weakly informative priors for most parameters (in line with prior choice recommendations by the stan developer group, see https://github.com/stan-dev/stan/wiki/Prior-Choice-Recommendations), with one exception: Given the presence of an effect of jerk difference on mentalising accuracy in our two previous studies [33, 34], we defined an informative prior for jerk difference, following a normal distribution centred around the effect observed in Schuster et al. [34]. All other priors were defined as a normal distribution for the intercept and all regression coefficients and a half-Cauchy distribution for residual and random effect variances (all prior distributions centred at 0). Each model was run for four sampling chains with a minimum of 4000 iterations each (1000 warm-up iterations). There were no indications of nonconvergence (all Rhat values = 1, no divergent transitions). All models discussed in this paper are listed in detail in the Supplementary Materials. Code and all data relevant to the main analyses are publicly available at https://osf.io/4zc7q/.

## Results

Results are structured as follows: We first report within-culture (UK, followed by Japan), and subsequently cross-culture analyses. Within each section, we first report confirmatory analyses relating to our pre-registered hypotheses, followed by exploratory analyses. For the purpose of readability and logical progression, the order in which confirmatory analyses are presented is different than that initially set out in the pre-registration.

### Within-culture analyses: UK

#### UK non-autistic adults exhibit poorer mentalising performance when interpreting animations created by autistic participants

**Fig. 1:**
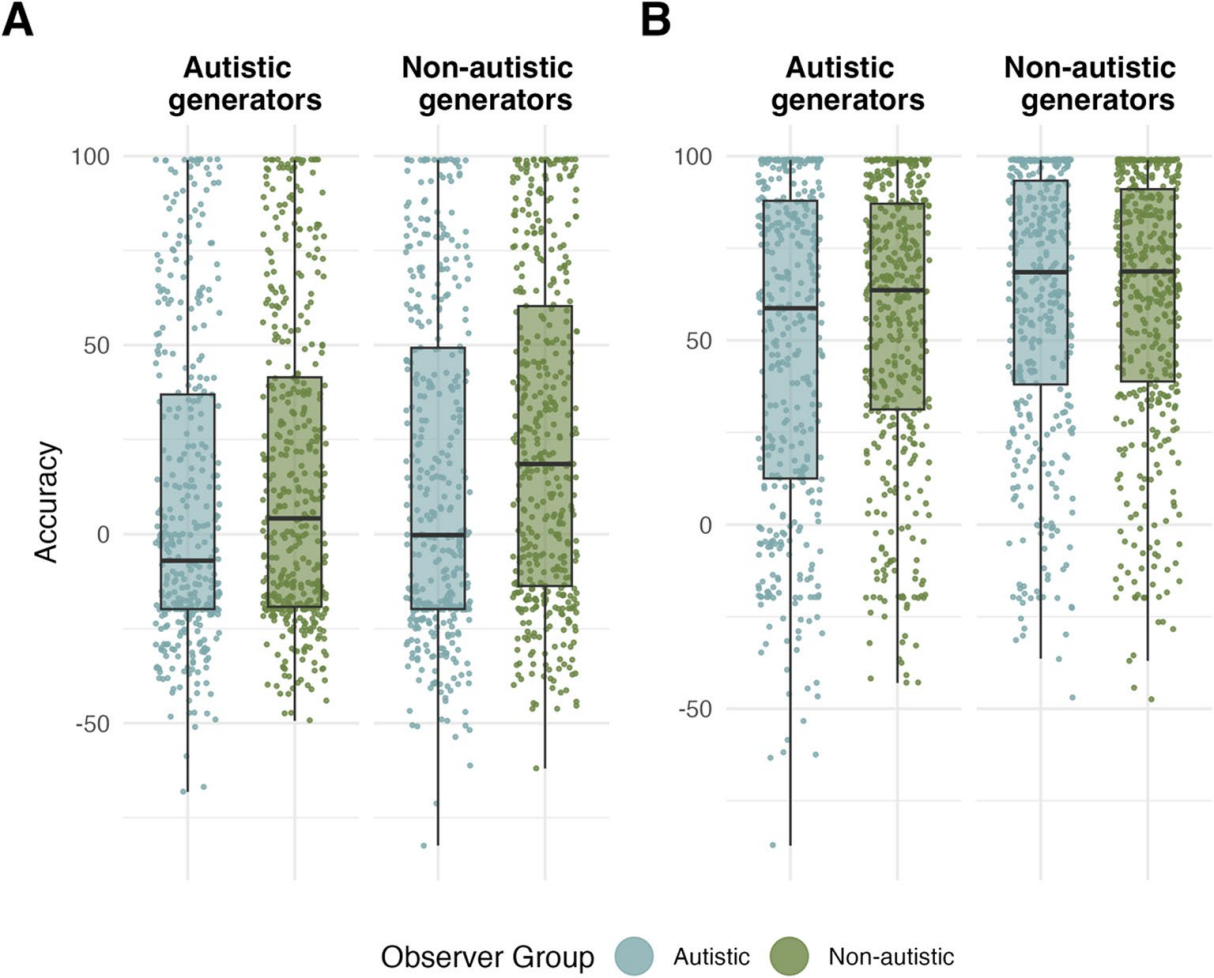
Accuracy by observer and generator group, UK data only for **A** mental-state animations. Autistic and non-autistic observers viewing animations created by autistic generators (left); non-autistic generators (right). **B** Non-mental state animations. Autistic and non-autistic observers viewing animations created by autistic generators (left); non-autistic generators (right). Central marks of box plots correspond to the median; outer hinges correspond to the first and third quartiles (25th and 75th percentiles). Upper and lower whiskers extend to largest and lowest values at most 1.5 * IQR of the hinge

We had two pre-registered hypotheses specifying our expectation that our results would replicate the findings of Edey et al. [26]. Hypothesis 2 predicted that non-autistic adults would struggle more to accurately interpret animations created by the autistic relative to their own generator group. Hypothesis 4 predicted that, as in Edey et al. [26], autistic participants would not show the same own-group benefit and thus exhibit comparable performance for animations generated by autistic and non-autistic groups. To test this, we used a Bayesian mixed-effects model (Model UK.1) with random intercepts and slopes for each predictor term varying by *subject ID* and *animation ID* (unique identifier for each animation), fitted to *accuracy* (see Animations Task) and the dummy coded predictors *generator group* (termed *gen* in effect indices; autistic [aut] vs non-autistic [non-aut]; reference level = non-aut) and *observer group* (termed *obs* in effect indices; autistic [aut] vs non-autistic [non-aut]; reference level = non-aut). To allow direct comparisons to the results in Edey et al. [26], this first model was fitted to accuracy in the *mental state* condition only. When only regarding mental state animations, there was a trend towards an effect for the contrast of autistic and non-autistic generators among non-autistic observers, indicating that overall, non-autistic adults tended to perform worse when interpreting animations created by autistic, relative to non-autistic adults (${E\mu }_{obs=non-aut,gen=autVSnon-aut}$ = − 7.06, CrI = [− 15.55, 1.40]; $P(E\mu <0)$ = 0.95; Note that, although strongly left-leaning, there is increased uncertainty around this effect). Post-hoc contrasts further showed that among autistic observers, there was no difference in performance between animations generated by the autistic or non-autistic group (${E\mu }_{obs=aut,gen=autVSnon-aut}$ = − 4.07, CrI = [− 14.00, 5.65]), supporting hypothesis 4. Finally, considering animations created by the other group, autistic and non-autistic adults performed comparably, as indicated by a lack of interaction between observer group and generator group (${E\mu }_{obs=autVSnon-aut,gen=autVSnon-aut}$ = 2.99, CrI = [− 4.31, 10.40], ${E\mu }_{obs=aut, gen=non-aut}$ = 14.30, CrI = [6.70, 21.90], ${E\mu }_{obs=non-aut, gen=aut}$ = 15.50, CrI = [8.14, 23.50]; Fig. 1A).

We then explored whether the results above depended on mental state condition by adding the dummy-coded factor *mental state* (mental vs non-mental; reference level = mental) to Model UK.1 (Model UK.2). Within non-autistic observers, this model showed a stronger, robust effect for generator group, with no interaction between mental state condition and generator group (mental state: ${E\mu }_{obs=non-aut,gen=autVSnon-aut}$ = − 8.60, CrI = [− 15.60, − 1.60], $P(E\mu <0)$ = 0.99; mental- vs non-mental state: ${E\mu }_{obs=non-aut,gen=autVSnon-aut}$ = 2.34, CrI = [− 7.11, 11.85], Fig. 1B), suggesting that the non-autistic observer group struggled to interpret animations generated by autistic adults, regardless of whether they conveyed mentalistic interactions or not, and thereby adding further support for hypothesis 2. Similarly, considering hypothesis 4, among autistic observers, there was no interaction between generator group and mental state condition, indicating that the lack of own-group benefit in this group did not depend on whether animations contained mentalistic interactions or not (${E\mu }_{obs=aut,gen=autVSnon-aut,non-mentalVSmental}$ = − 3.59, CrI = [− 15.13, 7.24]). Finally, this model showed no three-way interaction of generator group, observer group, and mental state (${E\mu }_{obs=autVSnon-aut,gen=autVSnon-aut,non-mentalVSmental}$ = − 5.90, CrI = [− 14.50, 2.77], suggesting that autistic and non-autistic participants showed comparable performance for other group-generated animations regardless of mental state condition.

#### In UK participants, movement similarity predicts accuracy for non-mental state animations generated by autistic participants only

Previous literature suggests that individuals use visuo-motor representations of their own movements to interpret the movements of others, and that higher kinematic similarity between agent and observer facilitates mentalising in the observer [32–34]. Although it is possible that there are fundamental differences in such putative motor simulation processes between autistic and non-autistic individuals (evidence is highly inconclusive; see Yates & Hobson [87]), a plausible alternative explanation for poorer mentalising performance in autistic individuals in previous studies employing animation tasks may be that there was little kinematic overlap between the (mostly experimenter-created) animation stimuli and the autistic participants’ own movements. If the latter notion is true, one should expect to observe a positive effect of movement similarity on mentalising accuracy among both autistic and non-autistic observers when stimuli are used which are representative of a broad spectrum of movement kinematics. Our hypothesis 5 thus predicted that we would observe an effect of movement similarity in the UK sample, irrespective of observer group.

**Fig. 2:**
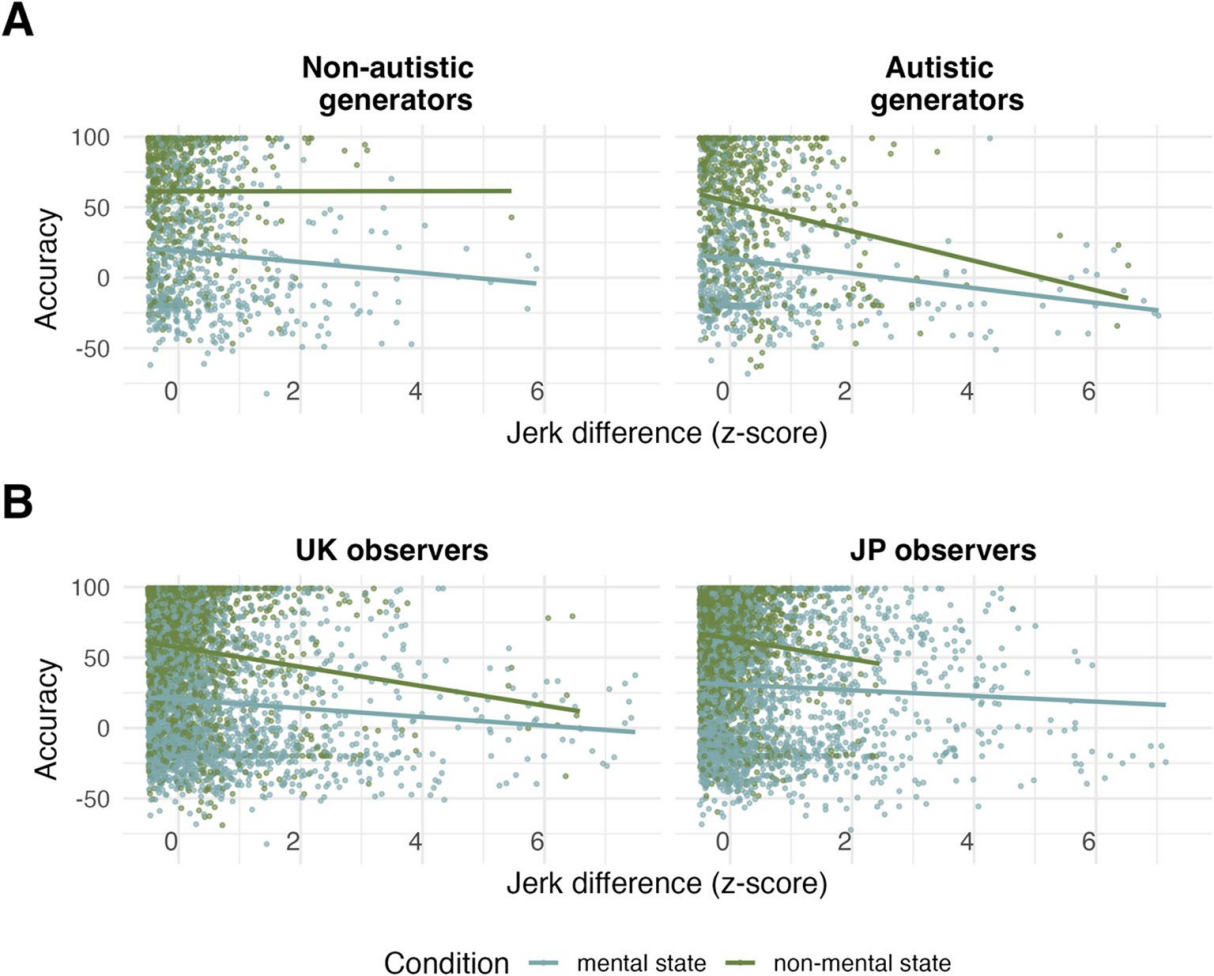
Effect of jerk difference by mental state and **A** generator group, UK data only (all participants viewing animations generated by non-autistic generators [left], autistic generators [right]), **B** observer culture (UK observers viewing animations generated by all participants [left], Japanese observers viewing animations generated by all participants [right])

*Jerk difference* was calculated by first subtracting the mean jerk (jerk was calculated as the third order non-null derivative of the raw positional data; for more details, see Schuster et al. [33]) of each video a participant rated from their own jerk values (derived from their animation of the same word), and then taking the absolute magnitude of those values. Thus, jerk difference serves as an index to observer-animator movement similarity, wherein lower values reflect higher jerk similarity. To test hypothesis 5, we ran a Bayesian mixed-effects model (Model UK.3) fitted to accuracy, jerk difference, the dummy-coded factor mental state (mental vs non-mental; reference level = mental) and their interaction with random intercepts for subject ID and animation ID and a random slope for all predictor terms varying by subject ID. This model revealed a negative effect of jerk difference (or a positive effect of movement similarity) for both mental- and non-mental state animations (${E\mu }_{jerkDiff, mental}$ = − 2.00, CrI = [− 3.96, − 0.03];

$P(E\mu <0)$ = 0.98; ${E\mu }_{jerkDiff, non-mentalVSmental}$ = 1.10, CrI = [− 2.80, 5.06]), suggesting that participants showed higher accuracy for animations wherein movements were more similar to their own. Adding the factor observer group to Model UK.3 (Model UK.4) resulted in a preserved effect for jerk difference (albeit with slightly increased uncertainty surrounding the effect: ${E\mu }_{jerkDiff, mental}$ = − 2.51, CrI = [− 5.11, 0.04]; $P(E\mu <0)$ = 0.97), and did not reveal an interaction between observer group and jerk difference (${E\mu }_{jerkDiff,obs=autVSnon-aut, mental}=$ 0.96, CrI = [− 2.31, 4.27]), suggesting that this effect did not depend on autism diagnosis and thereby supporting hypothesis 5. An exploratory model (Model UK.5) excluding observer group, but including the predictor generator group (autistic vs non-autistic; reference level = non-autistic) showed an interaction between jerk difference, generator group and mental state ($E\mu$ = − 6.07, CrI = [− 11.82, − 0.27], $P(E\mu <0)$ = 0.98), indicating a stronger negative effect of jerk difference for non-mental-, but not mental-state, animations, when those were created by the autistic-, relative to the non-autistic generator group (average marginal jerk difference trend for non-mental state, gen = autistic: $E\mu$ = − 4.01, CrI = [− 8.34, 0.28], gen = non-autistic: $E\mu$ = 3.75, CrI = [− 0.71, 8.00]; Fig. 2A).

In sum, within the British culture, non-autistic participants performed more poorly when interpreting animations generated by the autistic- relative to their own group, while autistic participants did not show such own-group advantage. Accuracy for other-group-generated animations was comparable across autistic and non-autistic adults. Finally, both autistic- and non-autistic observers showed an effect of movement similarity, but only for those animations generated by the autistic group.

### Within-culture analyses: Japan

#### Japanese non-autistic adults show comparable performance when interpreting animations generated by Japanese autistic and non-autistic adults

**Fig. 3:**
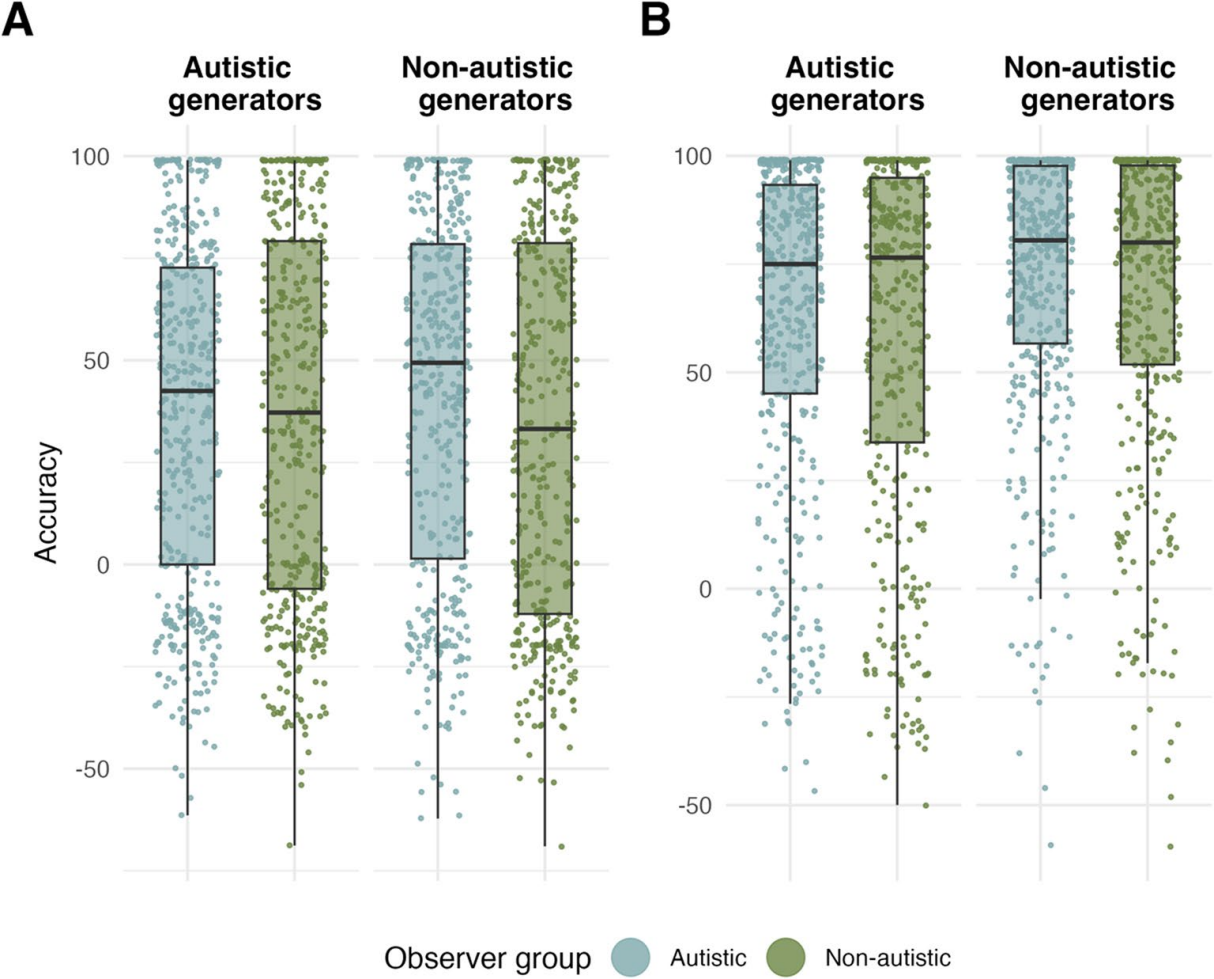
Accuracy by observer and generator group, Japanese (JP) data only for **A** mental-state animations. Autistic and non-autistic observers viewing animations created by autistic generators (left); non-autistic generators (right). **B** Non-mental state animations. Autistic and non-autistic observers viewing animations created by autistic generators (left); non-autistic generators (right). Central marks of box plots correspond to the median; outer hinges correspond to the first and third quartiles (25th and 75th percentiles). Upper and lower whiskers extend to largest and lowest values at most 1.5 * IQR of the hinge

Our pre-registered hypothesis 1 predicted that the bi-directional mentalising difficulties in non-autistic adults observed in Edey et al. [26] would be culturally independent, and stated that Japanese non-autistic adults would perform worse when interpreting animations generated by Japanese autistic, relative to non-autistic participants. To test this hypothesis, we ran a Bayesian mixed-effects model equivalent to Model UK.1 on Japanese data only (Model JP.1), fitted to accuracy and the dummy-coded predictors observer group (autistic vs non-autistic; reference level = non-autistic) and generator group (autistic vs non-autistic; reference level = non-autistic), for mental state animations only. Among non-autistic observers, the model revealed no effect of generator group (${E\mu }_{obs=non-aut,gen=autVSnon-aut}$ = 0.17, CrI = [− 8.61, 9.21]; Fig. 3A), suggesting that Japanese non-autistic adults performed equally well when interpreting animations generated by other Japanese autistic and non-autistic adults, and thus not supporting our hypothesis 1.

Exploratory post-hoc contrasts further showed that there was also no difference in performance among autistic observers between animations generated by the autistic or non-autistic group (${E\mu }_{obs=aut,gen=autVSnon-aut}$ = − 3.15, CrI = [− 12.61, 6.50]). Finally, regarding animations generated by the other group, there were no differences between autistic and non-autistic observers in the Japanese sample (${E\mu }_{obs=autVSnon-aut,gen=autVSnon-aut}$ = − 3.28, CrI = [− 9.89, 3.34], ${E\mu }_{obs=aut, gen=non-aut}$ = 38.40, CrI = [29.80, 47.30], ${E\mu }_{obs=non-aut, gen=aut}$ = 32.30, CrI = [23.40, 40.60], see Fig. 3A).

To test possible differences between mental and non-mental state animations, the dummy-coded variable mental state (mental vs non-mental; reference level = mental) was added to Model JP.1 (Model JP.2). There were no interactions between observer group or generator group and mental state in this model, indicating that the lack of group differences in mentalising performance in the Japanese sample did not depend on whether animations displayed mentalistic or purely action-based interactions (see Table S7, Fig. 3B).

#### Jerk difference does not predict mentalising accuracy within the Japanese sample

Based on prior literature suggesting culturally dependent display rules and a lower tendency to communicate internal states using body movements in collectivist cultures such as Japan [54], we expected that movement similarity will play a lesser role in mentalising within the Japanese sample. Our hypothesis 7 accordingly stated that the effect of jerk difference on mentalising accuracy will be less strong within Japanese, relative to UK participants. We first examined whether there was any effect of jerk difference present within the Japanese sample by fitting a Bayesian mixed-effects model with random intercepts for subject ID and animation ID and a random slope for all predictor terms varying by subject ID to accuracy and the covariate jerk difference, the dummy-coded factor mental state (mental vs non-mental; reference level = mental) as well as their two-way interaction (Model JP.3). This model revealed no effect of jerk difference for mental state animations (${E\mu }_{jerkDiff, mental}$ = 0.11, CrI = [− 2.27, 2.57]), and no interaction between jerk difference and mental state (${E\mu }_{jerkDiff, non-mentalVSmental}$ = − 1.34, CrI = [− 6.07, 3.27]), suggesting that within Japanese participants, movement similarity between agent and observer did not facilitate accurate interpretations of the animations. Two further exploratory models, one with the predictor observer group, and another with generator group (both factors: autistic vs non-autistic; reference level = non-autistic) added (Model JP.4, JP.5) showed no interactions between jerk difference and either of the groups (see Tables S9, S10), indicating that the lack of effect for movement similarity was independent of autism diagnosis.

Overall, among Japanese participants there were no differences in mentalising accuracy between autistic and non-autistic adults, and Japanese adults did not show any effect of movement similarity.

### Cross-culture analyses

#### There is a stronger negative trend for jerk difference among UK, compared to Japanese observers

To formally test our hypothesis 7 (see above), we fitted a new model (Model JPUK.1) to accuracy and the predictors jerk difference, mental state (mental vs non-mental; reference level = mental), and observer culture (JP vs UK; dummy-coded, reference level = UK), as well as all possible two- and three-way interactions, with random intercepts for subject ID and animation ID and a random slope for all predictor terms varying by subject ID. This model revealed a small negative trend for jerk difference for UK observers for both mental and non-mental state animations (mental state: $E\mu$ = − 1.31, CrI = [− 2.83, 0.19]; $P(E\mu <0)$ = 0.95; note there is increased uncertainty around this effect; non-mental vs mental state: $E\mu$ = 1.52, CrI = [− 0.91, 4.02]), and a three-way interaction between jerk difference, observer culture, and mental state (${E\mu }_{jerkDiff,obs=JPvsUK, non-mentalVSmental}$ = − 4.02, CrI = [− 7.61, − 0.53]; $P(E\mu <0)$ = 0.98). Post-hoc contrasts showed a more positive slope for jerk difference among Japanese observers for mental (${E\mu }_{jerkDiff,obs=JPvsUK}$ = − 1.94, CrI = [− 3.83, − 0.10], Fig. 2B) but not non-mental state (${E\mu }_{jerkDiff,obs=JPvsUK}$ = 2.09, CrI = [− 1.30, 5.59]) animations, suggesting a lower influence of movement similarity on mentalising performance within Japanese observers.

#### Japanese autistic adults show better mentalising performance than all UK participants

**Fig. 4:**
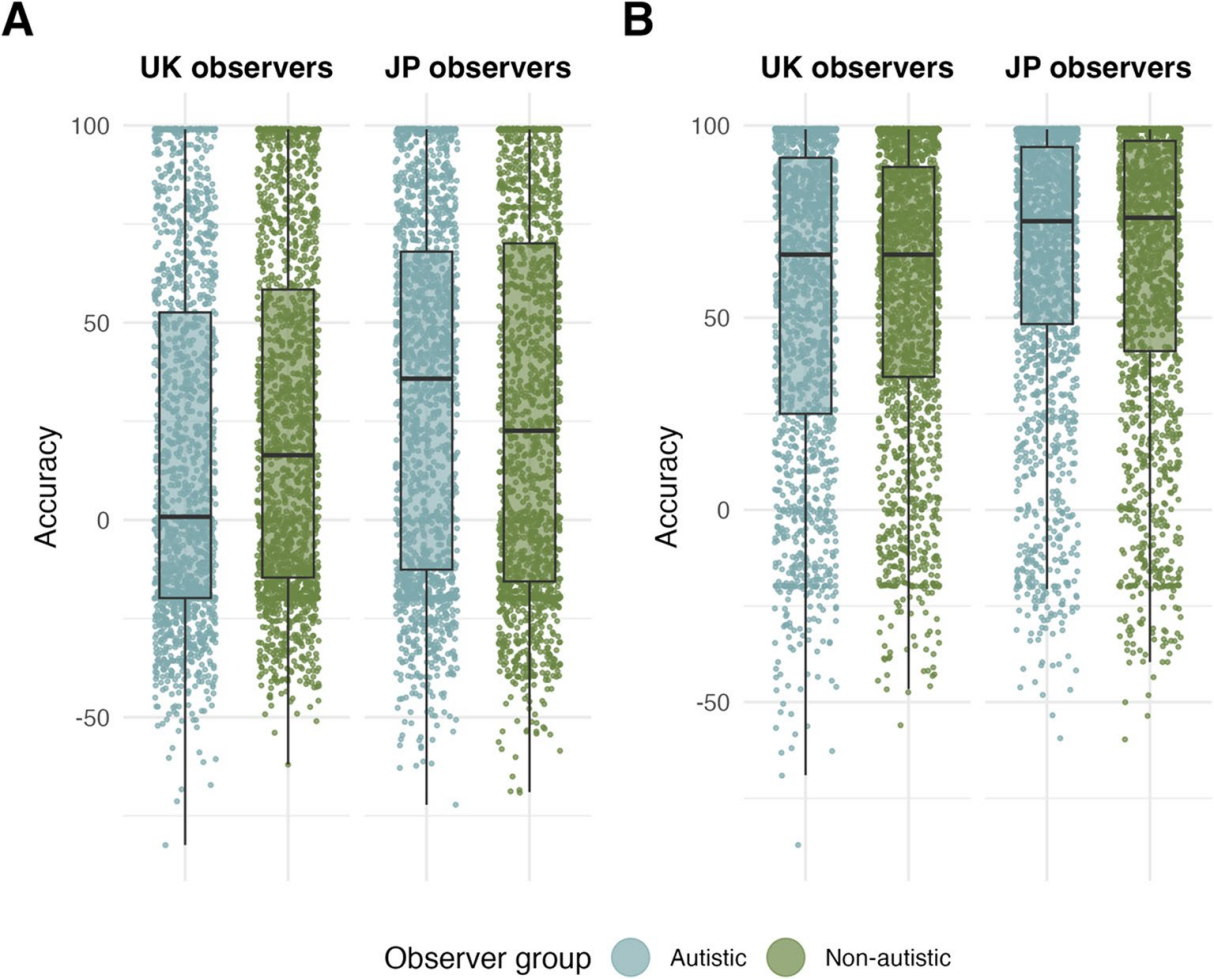
Accuracy by observer culture and observer group, for **A** mental-state animations. UK autistic and non-autistic observers viewing animations generated by all other participants (left); Japanese autistic and non-autistic observers viewing animations generated by all other participants (right). **B** Non-mental state animations. UK autistic and non-autistic observers viewing animations generated by all other participants (left); Japanese autistic and non-autistic observers viewing animations generated by all other participants (right). Central marks of box plots correspond to the median; outer hinges correspond to the first and third quartiles (25th and 75th percentiles). Upper and lower whiskers extend to largest and lowest values at most 1.5 * IQR of the hinge. UK = British culture, JP = Japanese culture

The latter model further revealed a main effect of observer culture, indicating that Japanese adults showed better mentalising performance relative to UK adults across mental- and non-mental state animations (mental state: ${E\mu }_{obs=JPvsUK}$ = 8.49, CrI = [3.26, 13.71], $P(E\mu <0)$ = 0.99; mental- vs non-mental state: ${E\mu }_{obs=JPvsUK}$ = 0.15, CrI = [− 5.53, 5.23]). To further test whether this difference in mentalising accuracy was driven by the lower mentalising accuracy observed in UK autistic observers, observer group (autistic vs non-autistic; reference level = non-autistic) was added to the previous model (JPUK.2). An interaction between observer culture and observer group (${E\mu }_{obs=JPvsUK,autVSnon-aut}$ = 9.65, CrI = [0.73, 18.51], $P\left(E\mu >0\right)$ = 0.98) and post-hoc contrasts showed that indeed, across both mental and non-mental state animations, Japanese non-autistic participants showed better mentalising performance relative to UK autistic (mental state: $E\mu$ = 9.35, CrI = [2.23, 16.53]), but not to UK non-autistic (mental state: $E\mu$ = 3.84, CrI = [2.58, 10.34]) adults. Contrasts further showed that Japanese autistic individuals exhibited better mentalising accuracy than both UK autistic (mental state: $E\mu$ = 13.57, CrI = [6.24, 20.56]) and non-autistic adults (mental state: $E\mu$ = 8.11, CrI = [0.41, 15.04], see Fig. 4). Note that we had no specific hypotheses about any cross-cultural differences in mentalising performance, therefore the latter results should be considered exploratory.

#### All autistic participants show higher accuracy for animations generated by Japanese autistic adults

One critical tenet of mismatch theories such as the double empathy problem is that there should be higher degrees of mutual understanding between individuals of the same neurotype. While indeed, cultural differences (e.g., different display rules [54]) may lead to mentalising difficulties between individiuals of two different cultures comparable to those observed between different neurotypes [26, 88, 89], autistic individuals have also been found to show a lower degree of social conformity [68–70]. Hypothesis 3 thus stated that we expected there to be no difference in performance between the two autistic groups when rating animations generated by autistic people of their own, relative to the other culture. To test this, a Bayesian mixed-effects model (Model JPUK.3) was fit to accuracy within autistic groups only and the dummy-coded predictors mental state (mental vs non-mental; reference level = mental), observer culture (JP vs UK, reference level = UK) and generator culture (JP vs UK, reference level = UK), as well as all possible interactions between predictors. Random intercepts and slopes were fit for all predictor terms varying by subject ID and animation ID. Model contrasts revealed an effect of generator culture for UK observers (mental state: ${E\mu }_{obs=UK,gen=JPvsUK}$ = 8.30, CrI = [0.46, 16.09], $P(E\mu >0)$ = 0.98; non-mental vs. mental state: ${E\mu }_{obs=UK,gen=JPvsUK}$ = − 3.12, CrI = [− 13.57, 7.20]), indicating that this autistic group performed better when animations were created by the other, relative to their own culture. There further was an interaction of observer and generator culture (mental state: ${E\mu }_{obs=JPvsUK,gen=JPvsUK}$ = 8.91, CrI = [2.72, 14.92], $(E\mu >0)$ = 0.99; non-mental vs. mental state: ${E\mu }_{obs=JPvsUK,gen=JPvsUK}$ = − 3.57, CrI = [− 11.70 4.67]), suggesting that Japanese autistic individuals interpreted animations of their own culture with higher accuracy than those generated by UK participants. A lack of interactions with mental state suggests that these results did not depend on whether animations displayed mentalistic or action-based interaction. In essence, both autistic groups performed better for animations that were created by Japanese autistic, relative to UK autistic generators, and thus do not lend support to hypothesis 3.

#### Autistic adults do not exhibit higher movement jerk than non-autistic adults

**Fig. 5:**
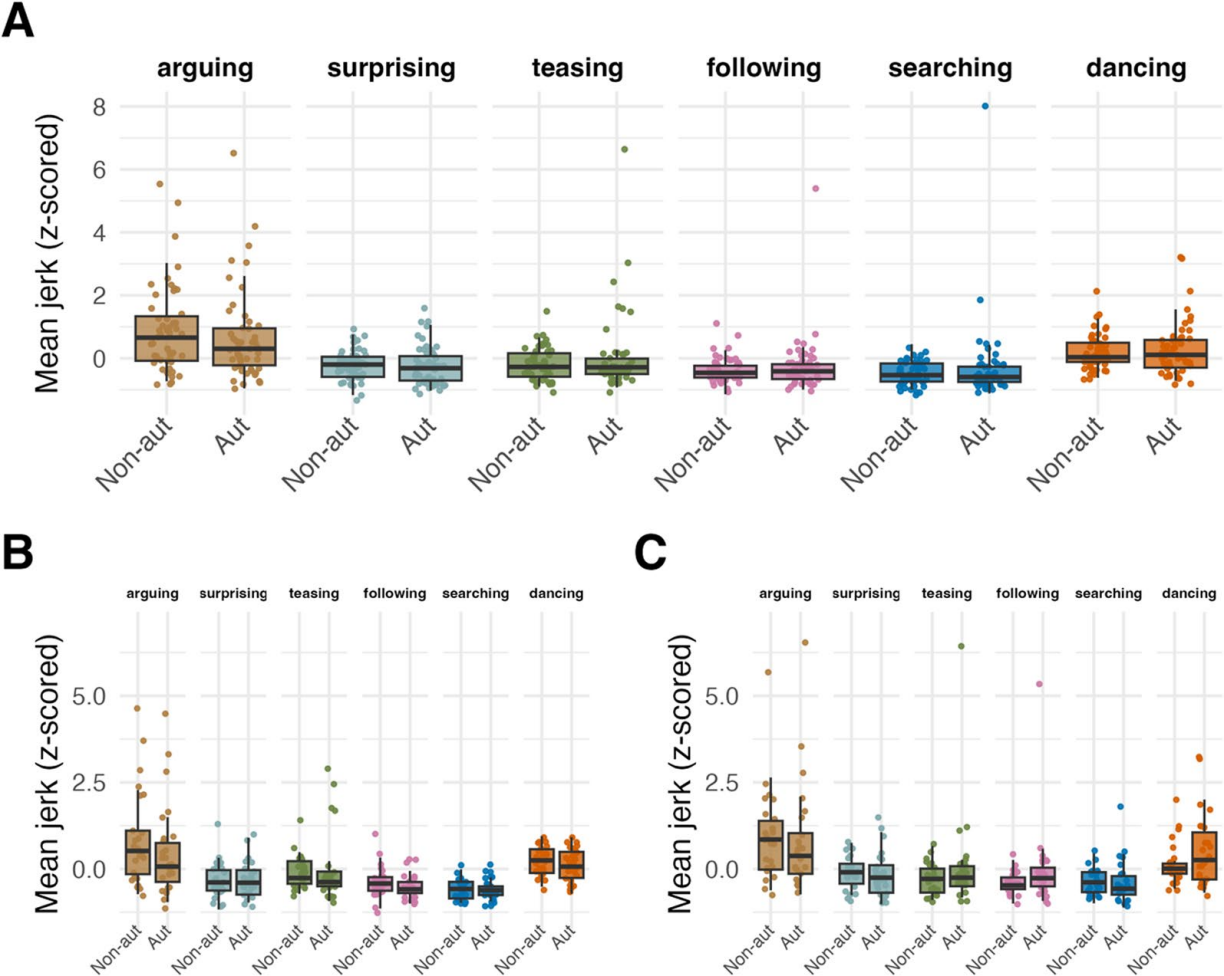
Average jerk across word types, by generator group, for **A** the whole sample, **B** the Japanese sub-sample and **C** the UK sub-sample. Central marks of box plots correspond to the median; outer hinges correspond to the first and third quartiles (25th and 75th percentiles). Upper and lower whiskers extend to largest and lowest values at most 1.5 * IQR of the hinge. Aut = Autistic, non-aut = non-autistic

**Fig. 6:**
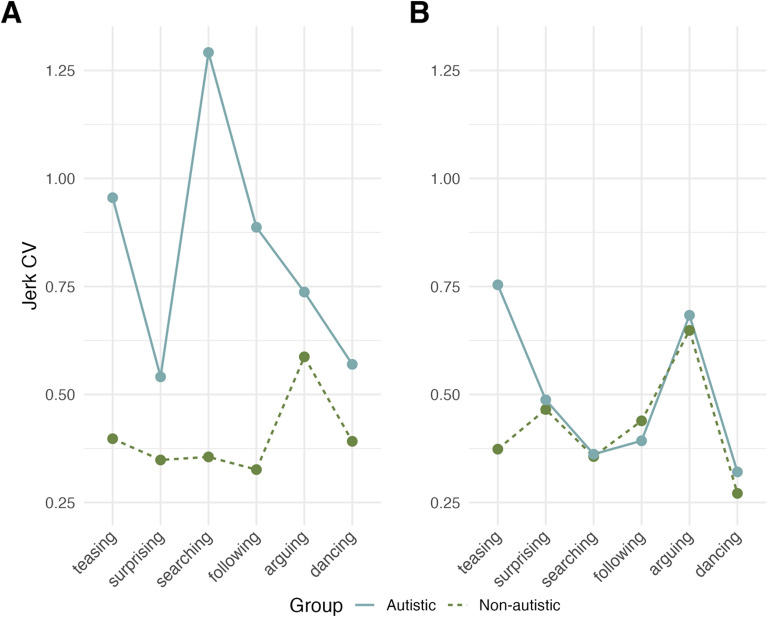
Coefficient of variability (CV) of jerk by generator group and word, for **A** UK and **B** Japanese participants

Finally, hypothesis 6 concerned differences in motor performance between autistic and non-autistic participants for both Japanese and British cultures. Given extensive literature suggesting that autistic individuals exhibit higher jerk across a range of movements (e.g., [26, 35, 36, 38]), we hypothesised that we would observe higher movement jerk in the autistic, relative to the non-autistic groups in our task, independent of culture. To test this, a Bayesian linear mixed-effects model was fit to mean jerk and the dummy-coded predictor variable group (autistic vs non-autistic, reference level = non-autistic), with random intercepts for subject ID and random slopes for the effect of group varying by subject ID (Model JPUK.4). This model showed no effect for group (${E\mu }_{AutVSnon-aut}$ = 0.06, CrI = [− 0.22, 0.34]), indicating no difference in mean jerk between autistic and non-autistic adults. To test the possibility that potential differences between groups depended on the type of word that was animated, *word* (levels arguing, surprising, teasing, following, searching, dancing; coded as deviation contrast), as well as the two-way interaction of word and group, were added as predictors to the previous model (Model JPUK.5). Model JPUK.5 revealed an interaction between the contrasts for ‘arguing’ and group, suggesting lower jerk values among autistic, relative to non-autistic participants for this word (${E\mu }_{jerk,autVSnon-aut, arguingVSmean}$= − 0.30, CrI = [− 0.61, − 0.00], $P(E\mu <0)$ = 0.98). Post-hoc contrasts showed that there was a smaller difference in jerk between the words teasing and arguing, and searching and arguing, for autistic compared to non-autistic generators (${E\mu }_{AutVSnon-aut,arguingVSteasing}$ = − 0.47, CrI = [− 0.86, − 0.09], ${E\mu }_{AutVSnon-aut,arguingVSsearching}$ = − 0.40, CrI = [− 0.80, − 0.02]; non-autistic: ${E\mu }_{arguingVSteasing}=$− 1.06, CrI = [− 1.33, − 0.80], ${E\mu }_{arguingVSsearching}=$− 1.35, CrI = [− 1.61, − 1.08]; autistic: ${E\mu }_{arguingVSteasing}=$− 0.59, CrI = [− 0.88, − 0.32], ${E\mu }_{arguingVSsearching}=$− 0.94, CrI = [− 1.25, − 0.65], Fig. 5). We further added the dummy-coded factor culture (JP vs UK, reference level = UK) to the latter model to confirm that, as hypothesised, there was no difference in jerk between the autistic groups of each culture (Model JPUK.6). In this new model, there was increased uncertainty around the original interaction of group and ‘arguing’ within the UK sample (${E\mu }_{AutVSnon-aut, arguingVSmean}$= − 0.34, CrI = [− 0.76, 0.10], $P(E\mu <0)$ = 0.94), no main effect for culture (${E\mu }_{JPvsUK}$ = − 0.05, CrI = [− 0.29, 0.19]) or group (${E\mu }_{AutVSnon-aut}$ = 0.20, CrI = [− 0.20, 0.61]), and no interaction between group and culture (${E\mu }_{ASCvsnon-ASC,JPvsUK}$ = 0.20, CrI = [− 0.19, 0.60]). Edey et al. [26] speculated that one possible reason for their observation of an own-group mentalising benefit in the non-autistic, but not the autistic group, may have been the higher levels of movement similarity they observed among their non-autistic participants. Given in the present study, we found the same lack of own-group benefit in the UK autistic group, we explored whether this group also exhibited higher movement variability. To this end, we first calculated a proxy for *jerk variability* as the coefficient of variation (CV) of jerk values within each culture and group for each of the words, resulting in 6 jerk variability values per culture and group. A mixed-effects model (JPUK.7) was fit to jerk variability, predicted by the two dummy-coded factors group (autistic vs non-autistic, reference level = non-autistic), culture (JP vs UK, reference level = UK), and their two-way interaction, with a random intercept for word. For UK generators, this model revealed a strong effect of group (${E\mu }_{gen=UK,autVSnon-aut}$ = 0.43, CrI = [0.21, 0.65], $P(E\mu <0)$ = 0.99), while there was no main effect of culture (${E\mu }_{JPvsUK}$ = 0.03, CrI = [− 0.20, 0.25]) and an interaction between group and culture (${E\mu }_{autVSnon-aut,JPvsUK}$ = − 0.36, CrI = [− 0.67, − 0.04], $P(E\mu <0)$ = 0.99), suggesting higher jerk variability among autistic generators in the UK, but not the Japanese sample (contrast for jerk variability within the Japanese sample: ${E\mu }_{gen=JP,autVSnon-aut}$ = 0.07, CrI = [− 0.15, 0.30]; Fig. 6).

Comparing British and Japanese cultures, our results show that, while there was no difference in mentalising performance between non-autistic adults, Japanese autistic participants interpreted animations with higher accuracy than both British groups. While British participants utilised their own movements to interpret the movements generated by others, Japanese participants did not show evidence for such mechanism. Furthermore, amongst autistic participants, animations generated by Japanese autistic adults were interpreted with higher accuracy than those made by UK participants. Finally, there was no difference in average movement jerk between autistic and non-autistic participants, however UK autistic participants showed higher movement variability relative to British non-autistic adults.

## Discussion

Over the past decade, the idea that communication challenges between autistic and non-autistic individuals may not be owed to mentalising difficulties present in the autistic partner alone, but rather be attributable to a two-way mismatch, has gained increasing attention in both the autism community and the scientific literature. Yet, to date little empirical data exists which directly support this theory. The primary objective of the present study, specified in pre-registered hypotheses 1 and 2 respectively, was therefore two-fold: First, we aimed to replicate the findings from a seminal study [26] which used a classical mentalising task—traditionally employed to reveal mentalising deficits in autistic populations—to demonstrate that non-autistic participants experience similar difficulties when interpreting social cues from autistic people. Second, we aimed to investigate the extent to which these findings are transferable to other, non-Western cultures. As expected, in our UK sample, non-autistic adults showed poorer mentalising performance for animations that were generated by the autistic, relative to those created by their own group, thus replicating the main finding reported by Edey et al. [26]. This result is further in agreement with other prior studies showing that non-autistic individuals struggle to read autistic people’s emotional expressions [89, 90], and aligns with predictions made by mismatch theories [14, 16], that non-autistic people struggle to understand autistic people in the same way as autistic people have difficulties understanding their non-autistic interaction partners.

Crucially however, results from Edey et al. [26], as well as from the present study, suggest that while social interactions may be more successful and mutual understanding greater among neurotypical people, the same may not apply for interactions between autistic individuals. While in our and Edey and colleagues’ studies both non-autistic groups showed higher mentalising performance for own-group, rather than other-group generated animations, the autistic groups did not show such own-group benefit. Based on a body of work suggesting that humans use internal models of their own actions when interpreting the actions of others [28, 29, 32–34], Edey et al. suggested that the own-group advantage among their non-autistic participants may have resulted from higher kinematic overlap between animation generators and observers within this group. They further speculated that a reason for the lack of own-group benefit within the autistic group may have been the increased kinematic variability observed in this group. Interestingly, while the animations generated by UK autistic adults in our study were on average not higher in jerk than those of non-autistic participants, they exhibited higher inter-subject variability in jerk. This raises the possibility that among UK autistic-generated animations, it was those with the highest differences in jerk relative to the grand mean which were most difficult to interpret for both observer groups and thus may have driven the effect of generator group. This idea is supported by the fact that we observed an effect of movement similarity (i.e., negative effect of jerk difference) on mentalising accuracy only for those animations that were generated by autistic people, with lower mentalising accuracy for those animations with the lowest kinematic overlap between observer and generator. Our findings of higher movement variability alongside a lack of own-group mentalising benefit within the autistic group are reminiscent of a study by Georgescu et al. [91], who found reduced interpersonal synchrony (operationalised as displacement between two time series of motion energy) in autistic and cross-neurotype dyads, relative to dyads of non-autistic people. They are further line with a large body of work which attests high heterogeneity across many levels of analysis [92], and idiosyncratic behavioural [90, 93] and neural [94, 95] patterns within studied samples of autistic individuals, with some researchers arguing that autism lacks biological and construct validity [96]. Findings like these raise the possibility that we may be wrongly averaging across unique brains, thus potentially leading to null results which mask informative data patterns. Accordingly, there may be not one, but many different [97], neurotypes relating to autism, which may also be why the present, and several previous [26, 91, 98] studies fail to find evidence for enhanced social interaction within autistic dyads (though see Crompton et al. [99], who found equally effective information transfer in autistic and non-autistic, and relatively worse communication in mixed chains of people).

In summary, within the UK sample, our data are consistent with our hypotheses 2 and 4, providing further evidence for the idea that mentalising difficulties may be bi-directional. Our findings thus align with the primary notion brought forward by mismatch theories [14, 16] and other recent accounts [12], emphasising the influence of individual experiences on mentalising processes. Critically, although such theories have thus far predicted that mentalising should be more successful between autistic people [14, 15], the present and past [26, 90–95, 98] findings suggest that experiences of (Western) autistic individuals may be too different, and perspective mismatches too pronounced, to support this prediction.

Our results further provide partial support for hypothesis 5, showing that higher kinematic overlap between animator and observer facilitated mentalising when the animator belonged to the autistic group, and thus adding to a line of evidence [27–34] suggesting that human observers utilise internal action models when inferring mental states from others’ movements. Importantly, there were no differences between autistic and non-autistic observers regarding the extent to which they made use of their own motor representations when interpreting others’ movements, contrasting prior evidence [100] in support of atypical mirror neuron system function in autism. However, our behavioural results do not necessarily preclude the existence of neural processing differences, as the hyperactivation observed in autism in some key mirror neuron nodes (such as the inferior frontal gyrus) in the latter meta-analysis may plausibly be a consequence of lower kinematic overlap between the autistic participants’ own movements and the stimuli used in the included studies.

Crucially, our analyses suggest that the latter findings are not transferable across different cultures. Contradicting our hypothesis 1, we did not observe any differences in mentalising performance between Japanese autistic and non-autistic observers, regardless of whether animations were generated by the autistic- or non-autistic group. There are several possible reasons why we did not replicate the results in Edey et al. [26] in the Japanese sample. One potentially influential factor may be camouflaging. Japan and other collectivist cultures are reported to show higher levels of autism-related stigma and lower acceptance of autism [101, 102], potentially leading to a higher motivation among individuals to camouflage their autistic traits. It is thus possible that, when creating the animations displaying mental state interactions, Japanese autistic adults (perhaps implicitly) mimicked the movements they thought non-autistic participants would display. Although prior research suggests comparatively lower camouflaging levels in Japan compared to many Western countries [102, 103], Japanese autistic adults may have self-reported lower camouflaging because they are less aware of exhibiting this behaviour. This is plausible as the collectivist nature of their culture demands high conformity to shared norms, and consequently a certain basic-level of concealing parts of one’s individual identity, from all members of society.

Alternatively, it is possible that relative to Western cultures, there is a smaller, or no, perspective mismatch between autistic and non-autistic people in Japan. Previous work has reported higher autistic traits in the general Japanese population compared to Western cultures such as the UK (mean AQ scores in Japan: 18.50–23.29 [73, 104] vs in the UK: 16.40 [64, 73]), which is replicated in the present study (see Table 2). Higher levels of autistic traits in Japan may at least in part be reflective of shared preferences, communication styles, and values between the Japanese culture and the general autistic population (for a comprehensive review see Atherton et al. [60]). For example, Japan is a ‘high uncertainty avoidance’ culture [105] (a characteristic which has been attributed to the high prevalence of natural disasters in this country), resulting in a higher reliance on structured environments, facilitated by rules and conventions which reduce ambiguity. Similarly, autism has been associated with a heightened intolerance for uncertainty [106], which in turn is linked to ‘insistence on sameness’ and a preference for routines [107]. If the latter notion is true, mutual mismatch theories would predict that it should be easier for Japanese autistic and non-autistic people to relate to each other, in which case the present results (i.e., a lack of difference in mentalising accuracy between autistic and non-autistic adults) would be a good reflection thereof, and consequently mentalising success in the animations task might be seen as a reasonable proxy for social communication and interaction abilities in the Japanese population.

However, there are several reasons why one may argue that this is not necessarily the case, and how the present results may rather illustrate how socio-cognitive measures developed in Western cultures are not fully transferable to other, non-Western cultures. While there is some variation across studies, most report higher prevalence rates of autism diagnoses in Japan compared to the UK [60, 66]. This suggests that the number of people who classify as needing support within the domains of social communication and interaction and restricted patterns of behaviour is higher in this country. Indeed, the Japanese culture has inherent characteristics which may be particularly difficult to navigate for autistic individuals. For instance, Japan is a so-called ‘high context’ culture [108, 109], where communication happens primarily through the use of non-explicit elements such as tone of voice, body language, and overall context—requiring its members to continually ‘read the air’ (a Japanese expression with a meaning similar to the English expression ‘read the room’). In contrast to this, autistic people tend to show difficulties with inferring implicit meaning, and a growing body of work indicates specific issues with integrating the context of (social) situations [110–113]. Moreover, as a highly collectivist culture [114], cultural norms and everyday life in Japan demand high levels of social conformity and perspective taking [115]. Regarding the latter, autistic individuals have been shown to struggle with shifting between one’s own and others’ visual [116] and cognitive [117] perspectives, potentially arising from difficulties with self-other control [118]. The former may present challenges to autistic people due to stronger discrepancies between their values and certain social norms (such as a preference for honesty [119] which may clash with the social imperative to conceal one’s true feelings if they are not in line with what is expected—in Japanese called ‘tatemae’ [120]), as well as a generally lower sensitivity to social influence [68–70]. Finally, the range of acceptable divergence from social norms may be narrower in Japan compared to Western cultures, and consequently deviations beyond it may be experienced as more aversive, which may be one of the reasons for comparatively higher levels of autism stigma in Japan [101]. Together, the latter points highlight that the degree of mismatch between the Japanese culture and the general autistic perspective may in fact be relatively high, despite some level of overlap in communication styles and preferences.

This raises the question why we did not observe performance differences between our Japanese autistic and non-autistic groups, and why Japanese autistic participants outperformed both UK observer groups. One potential explanation relates to the particular mentalising processes the animations task is thought to capture. The crucial difference to other existing mentalizing tasks (e.g., ones using social vignettes [121] or cartoons [122]) is that here participants are required to attribute mental states to the movements of geometric objects, that is, a certain degree of anthropomorphism (i.e., the ascription of human features to non-human entities [123]) is necessary to successfully perform the task. Intriguingly, anthropomorphism of non-living objects is deeply embedded in Japanese culture and is illustrated for instance by a high tendency to display everyday objects with human features (for example evident through the omni-presence of *yuru-kyara* or ‘mascots’). This propensity is presumed to stem from Shintoism—a Japanese religion which attributes animism to both living and non-living things—and it has been argued that this trait may be particularly strongly expressed within Japanese autistic individuals [60]. Notably, anthropomorphism and ToM are closely related and share the same neurobiological bases [123–125], and thus a cultural predisposition to anthropomorphise may be conducive to a mentalising advantage in the animations task. In Japanese autistic adults, a particularly strong interest in, and a lifetime engagement with, anthropomorphic stimuli such as anime (involving highly salient movement- and facial expression signals in conjunction) may have led to practice effects in interpreting the movements of non-human agents [60, 126, 127]. This notion resonates with a hypothesis posed by Koelkebeck et al. [49], who observed no behavioural differences, but lower mPFC activation when their Japanese, relative to Caucasian, participants interpreted animations. They speculated that this result may reflect a lower need to recruit the explicit mentalising system due to high attunement to implicit social meaning among Japanese individuals. Thus, a higher tendency to anthropomorphise in combination with superior implicit mentalising abilities due to demands owed to living in a high-context culture among Japanese participants may be why we observed such differences in mentalising performance between Japanese and British adults (though it should be noted that the cross-cultural contrast comparing Japanese and UK mentalising performances was exploratory and therefore replication is warranted). The question remains, then, why we did not observe any effect of movement similarity within the Japanese sample. Given culturally dependent display rules [54, 59], we initially expected our Japanese participants to show a lower tendency to openly express their emotions, and consequently to use movement cues to a lesser extent when inferring mental states. While our data support our hypothesis, one may argue that implicit mentalising necessitates successfully using movement cues, with shared action representations being a key route via which we can implicitly access others’ mental states [128]. It is possible that the kind of movement signals that aid Japanese individuals in mentalising are different to the patterns investigated in the present study. The Japanese culture places a strong emphasis on non-verbal communication (e.g., using multiple subtle variations of bowing [129]), and limited research suggests that the movements used to express one’s internal states differ between (Western and Eastern) cultures [130, 131]. Culturally specific body movement cues and differences in how these are used to understand others remain a much-understudied subject but are worth a more thorough investigation given potential consequences for the cultural sensitivity of research- and diagnostic tools which include the investigation of body movements (such as the ADOS-2).

In sum, elevated autism prevalence rates in Japan, as well as highly similar AQ scores among autistic people from Japanese and British cultures in this and prior [73] studies, suggest that the high performance of Japanese autistic people in the present mentalising task may not be a good proxy to individuals’ true social abilities, and thus poses the question whether the animations task has limited cultural transferability.

## Limitations

We note some limitations that need to be considered when interpreting the current results. First of all, although all efforts were made to ensure matched samples within and across cultures in terms of IQ, gender and age, this was not successful for all comparisons. We failed to match Japanese autistic and non-autistic groups for IQ, with the autistic group exhibiting a significantly lower IQ on average. We argue however, that since we observed no mentalising differences in this sample, this does not affect the interpretation of our results. In fact, our observation of a lack of mentalising differences in the presence of differences in IQ is in agreement with a line of prior research [132–134] suggesting that difficulties with mental state inference are independent of general reasoning abilities. In addition, British autistic and non-autistic groups were not matched for age, with autistic participants being significantly older than the non-autistic group. In contrast to prior work [135] indicating a covariation of higher-level socio-cognitive processes with age, our control analyses suggest no influence of participant age on any of the main effects observed (see Supplementary Results 1, Tables S18/S19). Furthermore, although this study aimed to investigate bi-directional difficulties in mentalising, on many occasions the differences we found were not specific to the mental state condition. Thus, we cannot say for sure that the present results reflect true differences in mentalising. In line with a large meta-analysis [25] also showing (slightly less pronounced) differences for non-mental state (i.e., goal-directed) animations between autistic and non-autistic observers, it is possible that animations tasks are primarily sensitive to differences in domain-general, lower- (e.g., motion processing [136]) or higher-level (e.g., central coherence [137]) cognitive processes, which may be underlying a vast array of social difficulties. Relatedly, animations tasks alongside other tests of ToM have been argued to have methodological limitations which may disadvantage autistic individuals in ways unrelated to ToM abilities [12]. This, as well as large heterogeneity within autistic samples, may be contributing to the generally poor construct- and predictive validity [4] of ToM tasks. Ultimately, the present task likely only taps into one of many aspects of the umbrella skill that is ToM. Future studies should compare a broader range of ToM tasks cross-culturally while accounting for potentially high heterogeneity within all considered populations by using sub-group analyses and data-driven or computational approaches. A final limitation of the present study concerns the characterisation of our autistic samples. Notably, the assumption that diagnostic tools lack cultural sensitivity presupposes a potential for misclassification of cases. As a consequence, one might question the sampling validity of our Japanese autistic participants. This illustrates a Catch-22 problem of cross-cultural research, where the future refinement of diagnostic tools is constrained by the cultural sensitivity of the present classification means. Yet, as all our Japanese autistic participants were receiving regular clinical care due to persistent difficulties with social communication, we consider the present sample as representative of the larger Japanese autistic population. Another factor potentially affecting sample equivalence are differences in recruitment strategies used in the two countries. Although highly similar AQ and IQ scores across the two autistic cohorts indicate equivalence in autistic traits and cognitive profiles, differing recruitment strategies in the two countries may have introduced sampling bias in dimensions not captured by these measures (e.g., relating to differences in day-to-day support needs between the two groups). To gain a more comprehensive picture of sample equivalence, future studies could benefit from including measures assessing adaptive functioning, such as the Vineland-3 (Vineland Adaptive Behavior Scales—Third Edition [138]). Lastly, due to shortages in qualified assessment staff, we were unable to obtain confirmatory research diagnoses (using the ADOS-2 [61] or the Autism Diagnostic Interview-Revised [ADI-R] [139]) for some of our autistic participants, specifically eight out of the 25 UK autistic participants, and three out of 25 Japanese autistic participants. Nonetheless, supplementary analyses (see Supplementary Results 2) confirm that, at least regarding autistic traits, our autistic participants were drawn from the same population: In both cultures, AQ scores did not differ significantly between those with and without a confirmatory research diagnosis.

## Conclusions

In conclusion, the present study provides new evidence to support a perspective shift in social cognition research, away from individual impairments towards the dynamic interplay between participants of social exchanges. Accordingly, mentalising challenges arise from relational mismatches, with body movement conveying mental state cues representing one dimension along which such mismatches can occur. Our results thus support a reframing of autism from a social communication disorder to a “description encompassing a broad range of developmental differences and experiences” (Milton [15], p. 1903). Future work should continue to systematically explore the origins of relational mismatches in social interactions, while using more nuanced definitions of divergent neurotypes (such as autism [97], but also schizophrenia [140] and attention deficit hyperactivity disorder [141] subtypes). A shift towards precision medicine and away from case–control paradigms will be impactful in expanding our current understanding of mismatch accounts of mentalising. Amongst other approaches, recent advancements in the field of AI present a powerful avenue for the development of communication systems where any interaction including individuals of diverging neurotypes can be dynamically accommodated.

Our study further highlights a potential lack of cultural transferability of a commonly used socio-cognitive task, calling to attention the need for greater cultural awareness in autism research. As a minimum, to increase awareness for the cultural dependency of research results and measures, future studies should discuss the generalisability of their findings to other cultures. Moreover, mismatch accounts that construe social interaction difficulties as a relational misalignment between interaction partners need to acknowledge how the greater collective structure (e.g., culture) that interactions are embedded in can shape and perhaps even reinforce the misalignment [142]. Accordingly, one necessary consequence of accepting the social model of disability is recognising that autism may be constructed differently within different cultures—a realisation which has led to calls for adapting towards a ‘socio-cultural model of disability [60], which situates definitions of autism within different cultural contexts. Some have even argued for classifying autism as a culture of its own with a shared ‘political and social identity’ [143]. Importantly, our findings—illustrating cultural biases towards Western social norms—further have implications for clinical practice. Such cultural biases are not only prevalent in the research literature, but also deeply embedded in diagnostic tools, such as the DSM-V and the ADOS-2 [60, 144]. While current knowledge on the cultural adaptability of autism diagnostic and screening tools is limited, there is some evidence to suggest a lack of applicability of at least some items of these tools to non-Western cultures, which as a consequence may result in underdiagnosis of cases, detrimentally impacting affected individuals’ lives [145–147]. Future work examining how autism may present differently in specific cultures is therefore needed to develop more tailored diagnostic measures which include culture as a frame of reference when assessing social interaction and communication skills.

## Supplementary Information

Additional file 1.

## Data Availability

Code and all data relevant to the main analyses are publicly available under https://osf.io/4zc7q/files/osfstorage.
