# Supplementary material for: A cross-cultural examination of bi-directional mentalising in autistic and non-autistic adults
Source: Mol Autism. 2025 May 14;16:29. doi: 10.1186/s13229-025-00659-z (PMC12076830; doi:10.1186/s13229-025-00659-z)
Supplement: Supplementary file 1 — Additional file 1. [file 13229_2025_659_MOESM1_ESM.pdf]

Supplementary Materials

A cross-cultural examination of bi-directional mentalising in autistic and non-autistic adults

Schuster B.A., Okamoto, Y., Takahashi, T., Kurihara, Y., Keating, C., Cook, J.L., Kosaka H., Ide, M., Naruse, H., Kraaijkamp, C., Osu, R.

**Population-level effects**	**Estimate**	**Error**	**95% CrI (lower)**	**95% CrI (upper)**
Intercept	22.52	3.57	15.52	29.42
GenGroupAut	-7.06	4.34	-15.55	1.40
ObsGroupAut	-8.25	3.65	-15.33	-1.06
GenGroupAut, obsGroupAut	3.01	3.73	-4.31	10.39

**Group-level effects**	**Estimate (SD)**	**Error**	**95% CrI (lower)**	**95% CrI (upper)**
Subject ID (intercept)	9.61	1.47	7.04	12.82
Subject ID (genGroupAut)	1.95	1.49	0.07	5.55
Subject ID (obsGroupAut)	2.54	2.02	0.09	7.50
Subject ID (genGroupAut, obsGroupAut)	3.03	2.34	0.12	8.63
Animation ID (intercept)	22.11	2.00	18.26	26.13
Animation ID (genGroupAut)	5.69	4.69	0.17	17.20
Animation ID (obsGroupAut)	9.52	3.42	1.74	15.53
Animation ID (genGroupAut, obsGroupAut)	3.62	2.92	0.14	10.97

Table S1. Model parameters for model UK.1.
Model formula: Accuracy ~ genGroup * obsGroup + (1 + genGroup * obsGroup || sub) + (1 + genGroup * obsGroup || animID). GenGroup = generator group, genGroupAut = generator group contrast aut vs. non-aut. ObsGroup = observer group, obsGroupAut = observer group contrast aut vs. non-aut. Sub = subject ID, animID = animation ID. Aut = autistic, non-aut = non-autistic.

Population-level effects	Estimate	Error	95% CrI (lower)	95% CrI (upper)
Intercept	24.22	3.12	18.05	30.32
GenGroupAut	-8.60	3.56	-15.60	-1.60
ObsGroupAut	-8.57	3.48	-15.37	-1.69
MentSt0	30.10	4.27	21.49	38.30
GenGroupAut, obsGroupAut	2.40	3.34	-4.18	8.84
GenGroupAut, mentSt0	2.34	4.84	-7.11	11.85
ObsGroupAut, mentSt0	7.27	4.89	-2.27	16.84
GenGroupAut, obsGroupAut, mentSt0	-5.90	4.41	-14.50	2.83

Group-level effects	Estimate (SD)	Error	95% CrI (lower)	95% CrI (upper)
Subject ID (intercept)	9.77	1.38	7.31	12.82
Subject ID (genGroupAut)	1.44	1.11	0.06	4.14
Subject ID (obsGroupAut)	2.45	1.93	0.10	7.19
Subject ID (mentSt0)	15.68	2.29	11.62	20.59
Subject ID (genGroupAut, obsGroupAut)	3.52	2.34	0.17	8.65
Subject ID (genGroupAut, mentSt0)	2.10	1.60	0.08	5.95
Subject ID (obsGroupAut, mentSt0)	4.51	3.77	0.15	13.99
Subject ID (genGroupAut, obsGroupAut, mentSt0)	3.33	2.60	0.14	9.81
Animation ID (intercept)	16.90	1.62	13.78	20.04
Animation ID (genGroupAut)	10.04	4.88	0.59	18.22
Animation ID (obsGroupAut)	7.30	2.65	1.13	11.71
Animation ID (mentSt0)	1.90	1.47	0.07	5.44
Animation ID (genGroupAut, obsGroupAut)	3.22	2.46	0.14	9.16
Animation ID (genGroupAut, mentSt0)	3.46	2.85	0.11	10.77
Animation ID (obsGroupAut, mentSt0)	2.16	1.66	0.09	6.29
Animation ID (genGroupAut, obsGroupAut, mentSt0)	2.87	2.27	0.11	8.40

Table S2. Model parameters for model UK.2.

Model formula: Accuracy ~ genGroup * obsGroup * mentSt + (1 + genGroup * obsGroup * mentSt || sub) + (1 + genGroup * obsGroup * mentSt || animID). GenGroup = generator group, genGroupAut = generator group contrast aut vs. non-aut; obsGroup = observer group, obsGroupAut = observer group contrast aut vs. non-aut. MentSt0 = contrast mental state vs. non-mental state. Sub = subject ID, animID = animation ID. Aut = autistic, non-aut = non-autistic.

**Population-level effects**	**Estimate**	**Error**	**95% CrI (lower)**	**95% CrI (upper)**
Intercept	17.00	2.42	12.33	21.72
MentSt0	32.56	3.53	25.41	39.31
JrkDiff	-2.00	1.00	-3.96	-0.03
MentSt0, jrkDiff	1.10	2.01	-2.80	5.06
**Group-level effects**	**Estimate (SD)**	**Error**	**95% CrI (lower)**	**95% CrI (upper)**
Subject ID (intercept)	10.51	1.43	7.99	13.55
Subject ID (mentSt0)	15.69	2.18	11.85	20.45
Subject ID (jrkDiff)	1.41	0.99	0.06	3.66
Subject ID (jrkDiff, mentSt0)	5.18	2.17	0.81	9.40
Animation ID (intercept)	19.33	1.11	17.25	21.60

Table S3. Model parameters for model UK.3.
Model formula: Accuracy ~ jrkDiff * mentSt + (1 + jrkDiff * mentSt || sub) + (1 | animID). MentSt = mental state, mentSt0 = contrast mental state vs. non-mental state. JrkDiff = variable & linear trend jerk difference. Sub = subject ID, animID = animation ID.

**Population-level effects**	**Estimate**	**Error**	**95% CrI (lower)**	**95% CrI (upper)**
Intercept	20.59	2.82	14.96	26.04
MentSt0	30.29	3.94	22.43	37.87
JrkDiff	-2.51	1.32	-5.11	0.04
ObsGroupAut	-7.57	3.24	-13.91	-1.13
MentSt0, jrkDiff	1.10	2.74	-4.29	6.51
MentSt0, obsGroupAut	5.32	4.51	-3.54	14.23
JrkDiff, obsGroupAut	0.96	1.66	-2.31	4.27
MentSt0, jrkDiff, obsGroupAut	-0.04	3.42	-6.70	6.64
**Group-level effects**	**Estimate (SD)**	**Error**	**95% CrI (lower)**	**95% CrI (upper)**
Subject ID (intercept)	9.89	1.45	7.34	13.00
Subject ID (mentSt0)	15.19	2.30	11.12	20.14
Subject ID (jrkDiff)	1.59	1.07	0.07	3.99
Subject ID (obsGroupAut)	3.15	2.38	0.13	8.87
Subject ID (mentSt0, jrkDiff)	4.22	2.38	0.26	9.02
Subject ID (jrkDiff, obsGroupAut)	1.80	1.35	0.08	5.04
Subject ID (mentSt0, obsGroupAut)	6.00	4.29	0.25	15.84
Subject ID (mentSt0, jrkDiff, obsGroupAut)	5.04	3.09	0.25	15.84
Animation ID (intercept)	19.31	1.12	17.20	21.64

Table S4. Model parameters for model UK.4.
Model formula: Accuracy ~ mentSt * jrkDiff * obsGroup + (1 + jrkDiff * mentSt * obsGroup || sub) + (1 | animID). MentSt = mental state, mentSt0 = contrast mental state vs. non-mental state. JrkDiff = variable & linear trend jerk difference. ObsGroup = observer group, obsGroupAut = observer group contrast aut vs. non-aut. Sub = subject ID, animID = animation ID. Aut = autistic, non-aut = non-autistic.

Population-level effects	Estimate	Error	95% CrI (lower)	95% CrI (upper)
Intercept	20.47	2.87	14.91	26.10
MentSt0	33.16	3.90	25.39	40.81
JrkDiff	-1.08	1.43	-3.90	1.75
GenGroupAut	-7.59	3.41	-14.21	-0.87
MentSt0, jrkDiff	4.82	2.56	-0.16	9.84
MentSt0, genGroupAut	-0.31	4.66	-9.39	8.70
JrkDiff, genGroupAut	-1.68	1.80	-5.26	1.82
MentSt0, jrkDiff, genGroupAut	-6.07	2.95	-11.82	-0.27
**Group-level effects**	**Estimate (SD)**	**Error**	**95% CrI (lower)**	**95% CrI (upper)**
Subject ID (intercept)	10.59	1.45	8.06	13.75
Subject ID (mentSt0)	15.79	2.22	11.89	20.54
Subject ID (jrkDiff)	1.45	1.02	0.06	3.74
Subject ID (genGroupAut)	1.72	1.27	0.06	4.72
Subject ID (mentSt0, jrkDiff)	4.73	2.27	0.41	9.25
Subject ID (jrkDiff, genGroupAut)	1.47	1.06	0.07	3.94
Subject ID (mentSt0, genGroupAut)	2.50	1.80	0.11	6.68
Subject ID (mentSt0, jrkDiff, genGroupAut)	2.47	1.82	0.10	6.79
Animation ID (intercept)	18.99	1.10	16.92	21.23

Table S5. Model parameters for model UK.5.

Model formula: Accuracy ~ mentSt * jrkDiff * genGroup + (1 + jrkDiff * mentSt * genGroup || sub) + (1 | animID). MentSt = mental state, mentSt0 = contrast mental state vs. non-mental state. JrkDiff = variable & linear trend jerk difference. GenGroup = generator group, genGroupAut = generator group contrast aut vs. non-aut. Sub = subject ID, animID = animation ID. Aut = autistic, non-aut = non-autistic.

**Population-level effects**	**Estimate**	**Error**	**95% CrI (lower)**	**95% CrI (upper)**
Intercept	32.04	4.36	23.43	40.61
GenGroupAut	0.17	4.55	-8.61	9.21
ObsGroupAut	6.30	4.46	-2.59	14.86
GenGroupAut, obsGroupAut	-3.28	3.40	-9.92	3.32

**Group-level effects**	**Estimate (SD)**	**Error**	**95% CrI (lower)**	**95% CrI (upper)**
Subject ID (intercept)	14.78	1.91	11.44	18.96
Subject ID (genGroupAut)	1.95	1.50	0.07	5.55
Subject ID (obsGroupAut)	3.15	2.57	0.12	9.58
Subject ID (genGroupAut, obsGroupAut)	2.43	1.95	0.09	7.24
Animation ID (intercept)	24.42	1.96	20.74	28.48
Animation ID (genGroupAut)	4.75	4.10	0.18	15.41
Animation ID (obsGroupAut)	3.34	2.42	0.14	8.92
Animation ID (genGroupAut, obsGroupAut)	3.54	2.75	0.14	10.16

Table S6. Model parameters for model JP.1.

Model formula: Accuracy ~ genGroup * obsGroup + (1 + genGroup * obsGroup || sub) + (1 + genGroup * obsGroup || animID). GenGroupAut = generator group contrast aut vs. non-aut. ObsGroupAut = observer group contrast aut vs. non-aut. Sub = subject ID, animID = animation ID. Aut = autistic, non-aut = non-autistic.

Population-level effects	Estimate	Error	95% CrI (lower)	95% CrI (upper)
Intercept	34.50	4.11	26.37	42.57
GenGroupAut	-1.97	3.84	-9.59	5.58
ObsGroupAut	5.89	4.36	-2.91	14.35
MentSt0	25.28	4.01	17.23	33.15
GenGroupAut, obsGroupAut	-2.78	2.88	-8.40	2.89
GenGroupAut, mentSt0	-1.30	5.01	-11.05	8.48
ObsGroupAut, mentSt0	-2.02	3.49	-8.84	4.93
GenGroupAut, obsGroupAut, mentSt0	0.42	3.90	-7.11	8.08

Group-level effects	Estimate (SD)	Error	95% CrI (lower)	95% CrI (upper)
Subject ID (intercept)	14.76	1.80	11.65	18.71
Subject ID (genGroupAut)	1.44	1.08	0.06	4.03
Subject ID (obsGroupAut)	2.98	2.47	0.10	9.17
Subject ID (mentSt0)	7.56	1.78	4.04	11.08
Subject ID (genGroupAut, obsGroupAut)	1.72	1.33	0.07	4.97
Subject ID (genGroupAut, mentSt0)	1.55	1.18	0.07	4.39
Subject ID (obsGroupAut, mentSt0)	3.15	2.37	0.13	8.75
Subject ID (genGroupAut, obsGroupAut, mentSt0)	2.04	1.59	0.08	5.97
Animation ID (intercept)	21.42	1.39	18.66	24.11
Animation ID (genGroupAut)	5.85	4.32	0.21	15.29
Animation ID (obsGroupAut)	2.03	1.51	0.08	5.62
Animation ID (mentSt0)	2.50	1.93	0.10	7.18
Animation ID (genGroupAut, obsGroupAut)	2.24	1.70	0.09	6.30
Animation ID (genGroupAut, mentSt0)	3.82	3.13	0.14	11.61
Animation ID (obsGroupAut, mentSt0)	1.57	1.20	0.06	4.50
Animation ID (genGroupAut, obsGroupAut, mentSt0)	1.90	1.49	0.07	5.55

Table S7. Model parameters for model JP.2.

Model formula: Accuracy ~ genGroup * obsGroup * mentSt + (1 + genGroup * obsGroup * mentSt || sub) + (1 + genGroup * obsGroup * mentSt || animID). GenGroupAut = generator group contrast aut vs. non-aut. ObsGroupAut = observer group contrast aut vs. non-aut. MentSt0 = contrast mental state vs. non-mental state. Sub = subject ID, animID = animation ID. Aut = autistic, non-aut = non-autistic.

**Population-level effects**	**Estimate**	**Error**	**95% CrI (lower)**	**95% CrI (upper)**
Intercept	35.86	3.08	29.85	41.97
MentSt0	23.26	3.13	17.00	29.35
JrkDiff	0.11	1.23	-2.27	2.57
MentSt0, jrkDiff	-1.34	2.39	-6.07	3.27
**Group-level effects**	**Estimate (SD)**	**Error**	**95% CrI (lower)**	**95% CrI (upper)**
Subject ID (intercept)	15.00	1.76	12.00	18.87
Subject ID (mentSt0)	6.98	1.74	3.57	10.44
Subject ID (jrkDiff)	4.26	1.02	2.31	6.31
Subject ID (jrkDiff, mentSt0)	2.90	2.16	0.12	8.06
Animation ID (intercept)	22.27	1.15	20.13	14.63

Table S8. Model parameters for model JP.3.

Model formula: Accuracy ~ jrkDiff * mentSt + (1 + jrkDiff * mentSt || sub) + (1 | animID). MentSt = mental state, mentSt0 = contrast mental state vs. non-mental state. JrkDiff = variable & linear trend jerk difference. Sub = subject ID, animID = animation ID.

Population-level effects	Estimate	Error	95% CrI (lower)	95% CrI (upper)
Intercept	33.54	3.67	26.16	40.65
MentSt0	23.82	3.41	17.18	30.47
JrkDiff	-0.35	1.49	-3.21	2.65
ObsGroupAut	4.62	4.24	-3.77	12.80
MentSt0, jrkDiff	-1.45	3.04	-7.43	4.59
MentSt0, obsGroupAut	-1.29	3.17	-7.49	5.04
JrkDiff, obsGroupAut	0.96	1.90	-2.78	4.70
MentSt0, jrkDiff, obsGroupAut	0.38	3.90	-7.27	7.99
**Group-level effects**	**Estimate (SD)**	**Error**	**95% CrI (lower)**	**95% CrI (upper)**
Subject ID (intercept)	14.88	1.81	11.76	18.87
Subject ID (mentSt0)	6.66	1.94	2.50	10.36
Subject ID (jrkDiff)	3.84	1.23	1.13	6.16
Subject ID (obsGroupAut)	3.05	2.50	0.12	9.30
Subject ID (mentSt0, jrkDiff)	2.81	2.11	0.11	7.81
Subject ID (jrkDiff, obsGroupAut)	2.43	1.65	0.11	6.10
Subject ID (mentSt0, obsGroupAut)	3.06	2.30	0.13	8.50
Subject ID (mentSt0, jrkDiff, obsGroupAut)	3.66	2.79	0.15	10.32
Animation ID (intercept)	22.31	1.15	20.13	24.64

Table S9. Model parameters for model JP.4.

Model formula: Accuracy ~ mentSt * jrkDiff * obsGroup + (1 + jrkDiff * mentSt * obsGroup || sub) + (1 | animID). MentSt = mental state, mentSt0 = contrast mental state vs. non-mental state. JrkDiff = variable & linear trend jerk difference. ObsGroup = observer group, obsGroupAut = observer group contrast aut vs. non-aut. Sub = subject ID, animID = animation ID. Aut = autistic, non-aut = non-autistic.

**Population-level effects**	**Estimate**	**Error**	**95% CrI (lower)**	**95% CrI (upper)**
Intercept	37.61	3.54	30.60	44.44
MentSt0	23.27	3.90	15.52	30.83
JrkDiff	-0.64	1.44	-3.46	2.19
GenGroupAut	-3.19	3.66	-10.35	4.00
MentSt0, jrkDiff	-2.38	3.09	-8.41	3.64
MentSt0, genGroupAut	-0.15	4.88	-9.78	9.39
JrkDiff, genGroupAut	1.66	1.76	-1.82	5.06
MentSt0, jrkDiff, genGroupAut	1.86	4.02	-6.05	9.79
**Group-level effects**	**Estimate (SD)**	**Error**	**95% CrI (lower)**	**95% CrI (upper)**
Subject ID (intercept)	15.02	1.77	11.95	18.88
Subject ID (mentSt0)	7.04	1.69	3.79	10.49
Subject ID (jrkDiff)	4.22	1.01	2.28	6.27
Subject ID (genGroupAut)	1.40	1.03	0.06	3.84
Subject ID (mentSt0, jrkDiff)	2.80	2.12	0.11	7.87
Subject ID (jrkDiff, genGroupAut)	1.55	1.16	0.06	4.28
Subject ID (mentSt0, genGroupAut)	1.52	1.17	0.06	4.37
Subject ID (mentSt0, jrkDiff, genGroupAut)	2.79	2.17	0.11	8.09
Animation ID (intercept)	22.20	1.14	20.04	24.52

Table S10. Model parameters for model JP.5.

Model formula: Accuracy ~ mentSt * jrkDiff * genGroup + (1 + jrkDiff * mentSt * genGroup || sub) + (1 | animID). MentSt = mental state, mentSt0 = contrast mental state vs. non-mental state. JrkDiff = variable & linear trend jerk difference. GenGroup = generator group, genGroupAut = generator group contrast aut vs. non-aut. Sub = subject ID, animID = animation ID. Aut = autistic, non-aut = non-autistic.

Population-level effects	Estimate	Error	95% CrI (lower)	95% CrI (upper)
Intercept	21.07	2.27	16.64	25.47
MentSt0	30.70	2.72	25.39	36.00
JrkDiff	-1.31	0.77	-2.83	0.19
ObsCultJP	8.49	2.64	3.26	13.71
MentSt0, jrkDiff	1.52	1.27	-0.91	4.02
MentSt0, obsCultJP	-0.15	2.74	-5.53	5.23
JrkDiff, obsCultJP	1.94	0.94	0.07	3.82
MentSt0, jrkDiff, obsCultJP	-4.02	1.79	-7.61	-0.53
**Group-level effects**	**Estimate (SD)**	**Error**	**95% CrI (lower)**	**95% CrI (upper)**
Subject ID (intercept)	12.06	1.21	9.69	14.52
Subject ID (mentSt0)	12.36	1.15	10.27	14.76
Subject ID (jrkDiff)	2.52	0.58	1.30	3.63
Subject ID (obsCultJP)	4.64	3.13	0.21	11.22
Subject ID (mentSt0, jrkDiff)	2.18	1.26	0.14	4.78
Subject ID (jrkDiff, obsCultJP)	1.27	0.85	0.06	3.14
Subject ID (mentSt0, obsCultJP)	2.13	1.61	0.09	5.99
Subject ID (mentSt0, jrkDiff, obsCultJP)	2.07	1.61	0.09	5.42
Animation ID (intercept)	21.70	0.74	20.32	23.24

Table S11. Model parameters for model JPUK.1.
Model formula: Accuracy ~ jrkDiff * mentSt * obsCult + (1 + jrkDiff * mentSt * obsCult || sub) + (1 | animID). MentSt = mental state, mentSt0 = contrast mental state vs. non-mental state. JrkDiff = variable & linear trend jerk difference. ObsCult = observer culture, obsCultJP = observer culture contrast JP vs UK. Sub = subject ID, animID = animation ID. JP = Japan.

Population-level effects	Estimate	Error	95% CrI (lower)	95% CrI (upper)
Intercept	21.07	2.27	16.64	25.47
MentSt0	30.70	2.72	25.39	36.00
JrkDiff	-1.31	0.77	-2.83	0.19
ObsCultJP	8.49	2.64	3.26	13.71
MentSt0, jrkDiff	1.52	1.27	-0.91	4.02
MentSt0, obsCultJP	-0.15	2.74	-5.53	5.23
JrkDiff, obsCultJP	1.94	0.94	0.07	3.82
MentSt0, jrkDiff, obsCultJP	-4.02	1.79	-7.61	-0.53
**Group-level effects**	**Estimate (SD)**	**Error**	**95% CrI (lower)**	**95% CrI (upper)**
Subject ID (intercept)	12.06	1.21	9.69	14.52
Subject ID (mentSt0)	12.36	1.15	10.27	14.76
Subject ID (jrkDiff)	2.52	0.58	1.30	3.63
Subject ID (obsCultJP)	4.64	3.13	0.21	11.22
Subject ID (mentSt0, jrkDiff)	2.18	1.26	0.14	4.78
Subject ID (jrkDiff, obsCultJP)	1.27	0.85	0.06	3.14
Subject ID (mentSt0, obsCultJP)	2.13	1.61	0.09	5.99
Subject ID (mentSt0, jrkDiff, obsCultJP)	2.07	1.61	0.09	5.42
Animation ID (intercept)	21.70	0.74	20.32	23.24

Table S12. Model parameters for model JPUK.2.

Model formula: Accuracy ~ jrkDiff * mentSt * obsCult * obsGroup + (1 + jrkDiff * mentSt * obsCult * obsGroup || sub) + (1 | animID). MentSt = mental state, mentSt0 = contrast mental state vs. non-mental state. JrkDiff = variable & linear trend jerk difference. ObsCult = observer culture, obsCultJP = observer culture contrast JP vs UK. JP = Japan. ObsGroup = observer group, obsGroupAut = observer group contrast aut vs. non-aut. Sub = subject ID, animID = animation ID. Aut = autistic, non-aut = non-autistic.

**Population-level effects**	**Estimate**	**Error**	**95% CrI (lower)**	**95% CrI (upper)**
Intercept	13.22	3.61	6.31	20.37
MentSt0	25.99	4.67	16.78	34.99
ObsCultJP	6.51	3.23	0.14	12.81
GenCultJP	8.30	4.01	0.46	16.09
MentSt0, obsCultJP	3.86	4.30	-4.53	12.31
MentSt0, genCultJP	-3.12	5.31	-13.57	7.20
ObsCultJP, genCultJP	8.91	3.10	2.72	14.92
MentSt0, obsCultJP, genCultJP	-3.57	4.20	-11.70	4.67
**Group-level effects**	**Estimate (SD)**	**Error**	**95% CrI (lower)**	**95% CrI (upper)**
Subject ID (intercept)	8.66	1.45	6.02	11.72
Subject ID (mentSt0)	12.55	1.96	9.04	16.72
Subject ID (obsCultJP)	3.01	2.28	0.11	8.41
Subject ID (genCultJP)	2.03	1.44	0.09	5.32
Subject ID (mentSt0, obsCultJP)	2.99	2.43	0.11	9.03
Subject ID (mentSt0, genCultJP)	2.24	1.67	0.09	6.22
Subject ID (obsCultJP, genCultJP)	2.06	1.57	0.08	5.77
Subject ID (mentSt0, obsCultJP, genCultJP)	2.38	1.90	0.10	7.15
Animation ID (intercept)	23.17	1.33	20.66	25.92
Animation ID (mentSt0)	3.05	2.45	0.12	9.20
Animation ID (obsCultJP)	3.35	2.19	0.18	8.04
Animation ID (genCultJP)	3.26	2.62	0.12	9.94
Animation ID (mentSt0, obsCultJP)	2.09	1.60	0.08	5.99
Animation ID (mentSt0, genCultJP)	3.13	2.60	0.10	9.76
Animation ID (obsCultJP, genCultJP)	3.57	2.55	0.12	9.32
Animation ID (mentSt0, obsCultJP, genCultJP)	2.55	2.02	0.11	7.57

Table S13. Model parameters for model JPUK.3.
Model formula: Accuracy ~ mentSt * obsCult * genCult + (1 + mentSt * obsCult * genCult || sub) + (1 + mentSt * obsCult * genCult || animID). MentSt = mental state, mentSt0 = contrast mental state vs. non-mental state. ObsCult = observer culture, obsCultJP = observer culture contrast JP vs UK. GenCult = generator culture, genCultJP = generator culture contrast JP vs. UK. Sub = subject ID, animID = animation ID. JP = Japan.

Population-level effects	Estimate	Error	95% CrI (lower)	95% CrI (upper)
Intercept	-0.03	0.06	-0.14	0.08
GroupAut	0.06	0.14	-0.22	0.34
**Group-level effects**	**Estimate (SD)**	**Error**	**95% CrI (lower)**	**95% CrI (upper)**
Subject ID (intercept)	0.23	0.08	0.05	0.38
Subject ID (groupAut)	0.81	0.11	0.62	1.04

Table S14. Model parameters for model JPUK.4.
Model formula: jerk ~ group + (1 + group || sub). GroupAut = group contrast aut vs. non-aut. Aut = autistic, non-aut = non-autistic. Sub = subject ID.

**Population-level effects**	**Estimate**	**Error**	**95% CrI (lower)**	**95% CrI (upper)**
Intercept	-0.03	0.06	-0.15	0.09
GroupAut	0.06	0.14	-0.23	0.33
Contrast teasing	-0.16	0.07	-0.30	-0.01
Contrast surprising	-0.19	0.07	-0.33	-0.06
Contrast searching	-0.44	0.07	-0.57	-0.30
Contrast following	-0.34	0.07	-0.47	-0.21
Contrast arguing	0.91	0.11	0.70	1.12
GroupAut, contrast teasing	0.17	0.11	-0.05	0.38
GroupAut, contrast surprising	-0.08	0.11	-0.29	0.13
GroupAut, contrast searching	0.10	0.12	-0.13	0.33
GroupAut, contrast following	0.05	0.10	-0.14	0.24
GroupAut, contrast arguing	-0.30	0.15	-0.61	-0.00
**Group-level effects**	**Estimate (SD)**	**Error**	**95% CrI (lower)**	**95% CrI (upper)**
Subject ID (intercept)	0.36	0.06	0.26	0.46
Subject ID (groupAut)	0.81	0.11	0.62	1.05
Subject ID (contrast teasing)	0.17	0.10	0.01	0.37
Subject ID (contrast surprising)	0.11	0.08	0.00	0.29
Subject ID (contrast searching)	0.10	0.07	0.00	0.26
Subject ID (contrast following)	0.08	0.06	0.00	0.23
Subject ID (contrast arguing)	0.58	0.08	0.43	0.76
Subject ID (groupAut, contrast teasing)	0.22	0.12	0.01	0.45
Subject ID (groupAut, contrast surprising)	0.33	0.14	0.05	0.59
Subject ID (groupAut, contrast searching)	0.46	0.12	0.21	0.68
Subject ID (groupAut, contrast following)	0.09	0.07	0.00	0.26
Subject ID (groupAut, contrast arguing)	0.18	0.13	0.01	0.47

Table S15. Model parameters for model JPUK.5.
Model formula: Jerk ~ group * cond + (1 + group * cond || sub). GroupAut = group contrast aut vs. non-aut. Aut = autistic, non-aut = non-autistic. Sub = Subject ID.

Population-level effects	Estimate	Error	95% CrI (lower)	95% CrI (upper)
Intercept	-0.00	0.09	-0.18	0.17
GroupAut	0.20	0.20	-0.19	0.60
CultJP	-0.05	-.12	-0.29	0.19
Contrast teasing	-0.26	0.10	-0.46	-0.06
Contrast surprising	-0.10	0.09	-0.29	0.09
Contrast searching	-0.33	0.09	-0.52	-0.15
Contrast following	-0.38	0.09	-0.57	-0.20
Contrast arguing	0.93	0.15	0.62	1.23
GroupAut, cultJP	-0.29	0.28	-0.85	0.27
GroupAut, contrast teasing	0.17	0.15	-0.13	0.47
GroupAut, contrast surprising	-0.26	0.16	-0.56	0.05
GroupAut, contrast searching	0.15	0.17	-0.23	0.41
GroupAut, contrast following	0.15	0.13	-0.12	0.41
GroupAut, contrast arguing	-0.34	0.22	-0.76	-0.10
CultJP, contrast teasing	0.21	0.15	-0.08	0.50
CultJP, contrast surprising	-0.18	0.13	-0.44	0.08
CultJP, contrast searching	-0.20	0.13	-0.46	0.06
CultJP, contrast following	0.07	0.13	-0.18	0.34
CultJP, contrast arguing	-0.04	0.21	-0.46	0.39
GroupAut, cultJP, contrast teasing	0.00	0.21	-0.42	0.42
GroupAut, cultJP, contrast surprising	0.35	0.22	-0.08	0.78
GroupAut, cultJP, contrast searching	0.01	0.23	-0.44	0.47
GroupAut, cultJP, contrast following	-0.19	0.19	-0.56	0.18
GroupAut, cultJP, contrast arguing	0.07	0.31	-0.55	0.66

Group-level effects	Estimate (SD)	Error	95% CrI (lower)	95% CrI (upper)
Subject ID (intercept)	0.37	0.05	0.27	0.48
Subject ID (groupAut)	0.80	0.11	0.60	1.03
Subject ID (contrast teasing)	0.19	0.11	0.01	0.39
Subject ID (contrast surprising)	0.12	0.08	0.01	0.30
Subject ID (contrast searching)	0.10	0.07	0.00	0.27
Subject ID (contrast following)	0.09	0.07	0.00	0.27
Subject ID (contrast arguing)	0.61	0.09	0.44	0.80
Subject ID (groupAut, contrast teasing)	0.22	0.12	0.01	0.46
Subject ID (groupAut, contrast surprising)	0.36	0.14	0.06	0.62
Subject ID (groupAut, contrast searching)	0.48	0.11	0.25	0.70
Subject ID (groupAut, contrast following)	0.10	0.07	0.00	0.27
Subject ID (groupAut, contrast arguing)	0.19	0.13	0.01	0.47

Table S16. Model parameters for model JPUK.6.
Model formula: Jerk ~ group * cult * cond + (1 + group * cond || sub). GroupAut = group contrast aut vs. non-aut. Cult = culture, CultJP = culture contrast JP vs. UK. Cond = word. Aut = autistic, non-aut = non-autistic. Sub = Subject ID.

Population-level effects	Estimate	Error	95% CrI (lower)	95% CrI (upper)
Intercept	0.40	0.09	0.21	0.59
GroupAut	0.43	0.11	0.21	0.65
CultJP	0.03	0.11	-0.20	0.25
GroupAut, cultJP	-0.36	0.16	-0.67	-0.04
**Group-level effects**	**Estimate (SD)**	**Error**	**95% CrI (lower)**	**95% CrI (upper)**
Cond (intercept)	0.10	0.09	0.00	0.31

Table S17. Model parameters for model JPUK.7.
Model formula: Jerk_cv ~ group * cult + (1 | cond). Jerk_cv = coefficient of variation of jerk. GroupAut = group contrast aut vs. non-aut. Cult = culture, cultJP = culture contrast JP vs. UK. Cond = word. Aut = autistic, non-aut = non-autistic.

Supplementary results 1: Analysis of effect of age on model UK.1/UK.2.

To test whether any of the observed effects in models UK.1 and UK.2 depended on age, 2 Bayesian mixed-effects models were run, with age and an interaction between all predictors and age added to the original models UK.1 and UK.2, respectively. As can be seen in Tables S18 and S19, there was no main effect of age, and no interactions between any of the predictors and age. While adding age resulted in increased uncertainty around the main effect of interest ($E\mu_{obs=non-aut,gen=autVSnon-aut}$, see contrast *GenGroupAut)* for model UK.1.2 (Table S18), it did not do so for model UK.2.2 (*GenGroupAut; GenGroupAut, mentSt*; Table S19).

Population-level effects	Estimate	Error	95% CrI (lower)	95% CrI (upper)
Intercept	22.19	3.91	14.49	29.74
GenGroupAut	-6.38	4.44	-15.13	2.26
ObsGroupAut	-9.79	4.54	-18.61	-0.80
Age	0.19	3.19	-6.04	6.55
GenGroupAut, obsGroupAut	5.03	4.38	-3.60	13.59
GenGroupAut, age	0.76	2.85	-4.81	6.44
ObsGroupAut, age	1.39	4.27	-6.94	9.81
GenGroupAut, obsGroupAut, age	0.94	3.89	-6.63	8.50

Group-level effects	Estimate (SD)	Error	95% CrI (lower)	95% CrI (upper)
Subject ID (intercept)	10.02	1.88	6.50	13.98
Subject ID (genGroupAut)	1.88	1.47	0.07	5.55
Subject ID (obsGroupAut)	3.01	2.52	0.11	9.38
Subject ID (age)	2.96	2.33	0.12	8.67
Subject ID (genGroupAut, obsGroupAut)	3.04	2.55	0.11	9.46
Subject ID (genGroupAut, age)	1.92	1.52	0.07	5.61
Subject ID (obsGroupAut, age)	3.00	2.45	0.11	9.02
Subject ID (genGroupAut, obsGroupAut, age)	2.59	2.20	0.09	8.24
Animation ID (intercept)	22.11	2.02	18.26	26.22
Animation ID (genGroupAut)	5.76	4.75	0.21	17.52
Animation ID (obsGroupAut)	6.76	3.99	0.33	14.47
Animation ID (age)	1.67	1.22	0.06	4.50
Animation ID (genGroupAut, obsGroupAut)	4.02	3.26	0.15	12.20
Animation ID (genGroupAut, age)	1.54	1.18	0.06	4.36
Animation ID (obsGroupAut, age)	1.51	1.15	0.06	4.26
Animation ID (genGroupAut, obsGroupAut, age)	1.83	1.43	0.07	5.35

Table S18. **Model parameters for control model UK1.2.**
Model formula: accuracy ~ genGroup * obsGroup * age + (1 + genGroup * obsGroup * age || sub) + (1 + genGroup * obsGroup * age || animID). GenGroup = generator group, genGroupAut = generator group contrast aut vs. non-aut. ObsGroup = observer group, obsGroupAut = observer group contrast aut vs. non-aut. Sub = subject ID, animID = animation ID. Aut = autistic, non-aut = non-autistic.

Population-level effects	Estimate	Error	95% CrI (lower)	95% CrI (upper)
Intercept	23.73	3.45	16.83	30.40
GenGroupAut	-8.02	2.65	-15.32	-0.94
ObsGroupAut	-9.97	4.50	-18.71	-0.95
MentSt0	30.06	4.53	20.92	38.66
Age	0.38	3.14	-5.84	6.59
GenGroupAut, obsGroupAut	4.14	3.86	-3.38	11.70
GenGroupAut, mentSt0	1.53	5.02	-8.13	11.40
ObsGroupAut, mentSt0	5.90	5.97	-6.03	17.53
GenGroupAut, age	0.52	2.52	-4.45	5.53
ObsGroupAut, age	1.06	4.16	-7.06	9.25
MentSt0, age	2.27	4.28	-6.26	10.45
GenGroupAut, obsGroupAut, mentSt0	-8.34	5.13	-18.34	1.55
GenGroupAut, obsGroupAut, age	1.33	3.44	-5.47	8.06
GenGroupAut, mentSt0, age	-1.47	3.48	-8.33	5.24
ObsGroupAut, mentSt0, age	0.56	5.67	-10.45	11.76
GenGroupAut, obsGroupAut, mentSt0, age	0.53	4.64	-8.73	9.58

Group-level effects	Estimate (SD)	Error	95% CrI (lower)	95% CrI (upper)
Subject ID (intercept)	10.55	1.82	7.28	14.46
Subject ID (genGroupAut)	1.37	1.06	0.06	3.93
Subject ID (obsGroupAut)	3.07	2.54	0.11	9.52
Subject ID (mentSt0)	15.29	3.88	5.64	22.37
Subject ID (age)	2.69	2.16	0.10	8.05
Subject ID (genGroupAut, obsGroupAut)	2.81	2.26	0.11	8.53
Subject ID (genGroupAut, mentSt0)	2.35	1.82	0.09	6.76
Subject ID (obsGroupAut, mentSt0)	7.25	6.30	0.23	23.00
Subject ID (genGroupAut, age)	1.53	1.19	0.06	4.43
Subject ID (obsGroupAut, age)	2.95	2.39	0.12	9.01
Subject ID (mentSt0, age)	5.22	4.51	0.17	16.70
Subject ID (genGroupAut, obsGroupAut, mentSt0)	4.27	3.51	0.15	13.08
Subject ID (genGroupAut, obsGroupAut, age)	2.60	2.14	0.09	7.98
Subject ID (genGroupAut, mentSt0, age)	2.37	1.88	0.09	7.05
Subject ID (obsGroupAut, mentSt0, age)	3.88	3.53	0.12	13.12
Subject ID (genGroupAut, obsGroupAut, mentSt0, age)	3.27	2.78	0.12	10.45
Animation ID (intercept)	16.59	1.68	13.44	19.95
Animation ID (genGroupAut)	11.58	4.85	0.96	19.35
Animation ID (obsGroupAut)	6.08	3.11	0.39	11.72
Animation ID (mentSt0)	1.93	1.50	0.07	5.56
Animation ID (age)	1.35	0.95	0.05	3.52
Animation ID (genGroupAut, obsGroupAut)	3.71	2.85	0.14	10.40

Animation ID (genGroupAut, mentSt0)	3.68	3.03	0.13	11.22
Animation ID (obsGroupAut, mentSt0)	2.49	1.95	0.09	7.23
Animation ID (genGroupAut, age)	1.35	1.00	0.06	3.74
Animation ID (obsGroupAut, age)	1.25	0.91	0.05	3.43
Animation ID (mentSt0, age)	1.33	0.98	0.05	3.63
Animation ID (genGroupAut, obsGroupAut, mentSt0)	3.36	2.74	0.13	10.23
Animation ID (genGroupAut, obsGroupAut, age)	1.46	1.11	0.05	4.08
Animation ID (genGroupAut, mentSt0, age)	1.91	1.41	0.08	5.21
Animation ID (obsGroupAut, mentSt0, age)	1.51	1.15	0.06	4.30
Animation ID (genGroupAut, obsGroupAut, mentSt0, age)	2.00	1.55	0.07	5.65

Table S19. **Model parameters for control model UK2.2.**
Model formula: accuracy ~ genGroup * obsGroup * mentSt * age + (1 + genGroup * obsGroup * mentSt * age || sub) + (1 + genGroup * obsGroup * mentSt * age || animID). GenGroup = generator group, genGroupAut = generator group contrast aut vs. non-aut; obsGroup = observer group, obsGroupAut = observer group contrast aut vs. non-aut. MentSt = mental state, mentSt0 = contrast mental state vs. non-mental state. Sub = subject ID, animID = animation ID. Aut = autistic, non-aut = non-autistic.

Supplementary results 2: Analysis of differences in autistic traits between individuals with and without a confirmatory research diagnosis

To investigate a potential sampling bias induced by the fact that some of our autistic participants did not have their diagnosis confirmed by an independent researcher, we conducted a Welch's independent sample t-test between individuals with and those without a confirmatory diagnosis for UK and Japanese autistic samples. This test yielded no differences between the UK or the Japanese autistic participants (UK: $t(7) = 2.36$, $p = 0.689$; Japan: $t(2) = 4.30$, $p = 0.950$).
